# Targeted disruption of the *BCR-ABL* fusion gene by Cas9/dual-sgRNA inhibits proliferation and induces apoptosis in chronic myeloid leukemia cells: Cas9/Dual-sgRNA targeting of the BCR-ABL fusion gene

**DOI:** 10.3724/abbs.2023280

**Published:** 2024-02-28

**Authors:** Jianling Zeng, Xinquan Liang, Lili Duan, Fenghua Tan, Liujie Chen, Jiayao Qu, Jia Li, Kai Li, Dixian Luo, Zheng Hu

**Affiliations:** 1 Translational Medicine Institute the First People’s Hospital of Chenzhou Hengyang Medical School University of South China Chenzhou 423000 China; 2 The First Affiliated Hospital of Xiangnan University Chenzhou 423000 China; 3 National & Local Joint Engineering Laboratory for High-through Molecular Diagnosis Technology the First People’s Hospital of Chenzhou Chenzhou 423000 China; 4 Department of Laboratory Medicine Huazhong University of Science and Technology Union Shenzhen Hospital (Nanshan Hospital) Shenzhen 518000 China; 5 National Engineering Research Center of Personalized Diagnostic and Therapeutic Technology Hunan University of Chinese Medicine Changsha 410208 China

**Keywords:** CRISPR/Cas9, dual sgRNA, *BCR-ABL*, chronic myeloid leukemia (CML), cell proliferation, apoptosis

## Abstract

The *BCR-ABL* fusion gene, formed by the fusion of the breakpoint cluster region protein ( *BCR*) and the Abl Oncogene 1, Receptor Tyrosine Kinase ( *ABL*) genes, encodes the BCR-ABL oncoprotein, which plays a crucial role in leukemogenesis. Current therapies have limited efficacy in patients with chronic myeloid leukemia (CML) because of drug resistance or disease relapse. Identification of novel strategies to treat CML is essential. This study aims to explore the efficiency of novel CRISPR-associated protein 9 (Cas9)/dual-single guide RNA (sgRNA)-mediated disruption of the *BCR-ABL* fusion gene by targeting *BCR* and *cABL* introns. A co-expression vector for Cas9 green fluorescent protein (GFP)/dual-BA-sgRNA targeting *BCR* and *cABL* introns is constructed to produce lentivirus to affect *BCR-ABL* expression in CML cells. The effects of dual-sgRNA virus-mediated disruption of *BCR-ABL* are analyzed via the use of a genomic sequence and at the protein expression level. Cell proliferation, cell clonogenic ability, and cell apoptosis are assessed after dual sgRNA virus infection, and phosphorylated BCR-ABL and its downstream signaling molecules are detected. These effects are further confirmed in a CML mouse model via tail vein injection of Cas9-GFP/dual-BA-sgRNA virus-infected cells and in primary cells isolated from patients with CML. Cas9-GFP/dual-BA-sgRNA efficiently disrupts *BCR-ABL* at the genomic sequence and gene expression levels in leukemia cells, leading to blockade of the BCR-ABL tyrosine kinase signaling pathway and disruption of its downstream molecules, followed by cell proliferation inhibition and cell apoptosis induction. This method prolongs the lifespan of CML model mice. Furthermore, the effect is confirmed in primary cells derived from patients with CML.

## Introduction

Characteristic fusion genes formed by translocation after a chromosome break are common in cancer, especially in leukemia. The abnormal expression and amplification of these fusion genes are the main causes of leukemia onset and progression. More than 90% of patients with chronic myeloid leukemia (CML) have the characteristic Philadelphia chromosome (translocation of chromosomes 9 and 22), forming the fusion gene *BCR-ABL* [encoding breakpoint cluster region protein (BCR)-Abl Oncogene 1, Receptor Tyrosine Kinase (ABL)]. The most common chromosomal breaks in patients with CML occur in the 13th or 14th intron of the *BCR* gene and the 1st intron of the *cABL* gene, forming the so-called fusion types b2a2 and b3a2, both of which lead to the p210 oncogenic fusion protein. The expressed fusion protein has abnormally increased tyrosine kinase activity, resulting in accelerated cell proliferation, inhibition of apoptosis, and adhesion defects [1].

Small molecule tyrosine kinase inhibitors (TKIs, *e*. *g*., Gleevec, Dasatinib, Nilotinib, and Bosutinib) inhibit the tyrosine kinase activity of fusion proteins to block signal transduction and lead to leukemia cell apoptosis and cell cycle arrest [ 2‒ 4]. In the clinic, these drugs achieve high remission rates, especially in patients with CML, and efficiently prolong patient survival. However, TKI therapy acts only on the expressed product of the fusion gene (the fusion protein) rather than the fusion gene itself and thus cannot eliminate pathogenic factors, limiting the ability of these drugs to cure leukemia. Treatment of CML using these drugs has several problems, such as poor efficacy in patients with progressive disease, side effects (such as cardiotoxicity) after long-term drug use, easy development of drug resistance, relapse, and inability to completely cure the disease [ 5‒ 7].

Clustered regularly interspaced short palindromic repeats (CRISPR)/CRISPR-associated protein 9 (Cas9) is a powerful gene-editing tool that can edit intracellular genes by destruction, deletion, point mutation, or mutation repair and has greatly promoted basic biomedical research [ 8‒ 12]. CRISPR/Cas9 technology has also shown great application potential in the treatment of diseases. The development and refinement of CRISPR/Cas9 gene editing technology provides potential solutions for treating more genetic diseases [ 13‒ 18].

The CRISPR/Cas9 system has been widely used for targeted genome modifications *in vivo* and *in vitro* in mammals, including gene knockout, gene mutation correction, gene insertion, and homology-directed repair [ 19, 20]. The *BCR-ABL* fusion gene is the oncogenic cause of CML pathogenesis; therefore, theoretically, knockout or disruption of *BCR-ABL* would ablate the production of the BCR-ABL fusion protein, which would likely cure CML. Several previous studies have reported that the CRISPR/Cas9 system can efficiently edit fusion genes in cancer cells. CRISPR/Cas9 technology has already been used to target *BCR-ABL* to prevent its possible oncogenic effects on leukemia cells [ 21, 22]. CRISPR RNA-guided *Fok*I nuclease and CRISPR/Cas9-directed gene trapping have also been used for the targeted disruption of *BCR-ABL* in K562 cells (a human CML-derived cell line) [ 23, 24]. However, these studies have limitations, either by targeting the *BCR-ABL* fusion coding sequence, which is not the true genomic sequence of *BCR-ABL*, or by ensuring that the resulting gene editing system is too large to be able to efficiently transduce into leukemia cells [ 21‒ 24].

The CRISPR/Cas9 system functions through a single guide RNA (sgRNA) that guides Cas9 to accurately cut gene-specific sites and activates cells to repair DNA through nonhomologous end joining (NHEJ), causing base insertions or deletions, resulting in frameshift mutations that effectively ablate gene expression. Thus, if Cas9 is guided to cut two sites in a sequence by two sgRNAs, a large DNA fragment can be deleted, leading to easier and more efficient knockout of the target gene. Herein, we focused on the P210 *BCR-ABL* fusion gene (including two subtypes, b3a2 and b2a2), which is the most common type in patients with CML and is shared by some patients with acute lymphoblastic leukemia (ALL) [25]. In contrast to the findings of previous reports, to disrupt the *BCR-ABL* gene, we designed two sgRNAs that target *BCR* and *cABL* introns, directing Cas9 to cut the two loci. A similar strategy was recently reported [26].

In the present study, our data showed that the genomic sequence of *BCR-ABL* was almost completely eliminated by this strategy, and the level of the BCR-ABL fusion protein was reduced significantly, leading to the inhibition of CML cell line proliferation and increased cell apoptosis *in vitro* and *in vivo*. The same effect was observed in primary cells isolated from patients with CML. These results prove the feasibility of using Cas9/dual-sgRNA targeting of the *BCR-ABL* fusion for gene therapy in CML.

## Materials and Methods

### Cell culture

The human CML cell lines K562 and KBM5 and the human renal epithelial cell line HEK293T were purchased from the American Type Culture Collection (ATCC; Manassas, USA). K562 cells were cultured in Roswell Park Memorial Institute (RPMI) 1640 medium (Gibco, Grand Island, USA). HEK293T and KBM5 cells were maintained in Dulbecco’s modified Eagle’s medium (DMEM; Gibco). All cell media were supplemented with 1% GlutaMAX (Gibco), 10 mg/L antibiotics (penicillin and streptomycin; P/S; Gibco) and 10% fetal bovine serum (FBS; Gibco). The cells were cultured at 37°C in a 5% CO _2_ and 5% O _2_ atmosphere in a humidified incubator. All the cell lines were periodically tested for mycoplasma contamination.

### DNA isolation and sequencing

Genomic DNA was extracted from the cultured cells using a DNA isolation kit (Tiangen, Beijing, China). Briefly, targeted gene sequences were amplified using premix LA Taq (TaKaRa, Shiga, Japan). The amplicons were separated using agarose gel electrophoresis, and the target bands were extracted and purified using a GEL/PCR Purification kit (Tiangen). A spectrophotometer was used to measure the DNA concentration. The PCR products were subsequently sequenced for verification.

### sgRNA design and T7E1 assay

The sgRNAs targeting the *BCR* or *cABL* genes were designed using the online CRISPR-sgRNA Design tool ( http://chopchop.cbu.uib.no/). The sgRNAs chosen were based on their high specificity rank and low potential off-target score [27]. The target sites and sgRNA sequences are listed in the supplemental materials ( Supplementary Table S1). Each sgRNA was cloned and inserted into the pCS2CMV-Cas9-IRES-GFP vector to construct a Cas9, GFP (green fluorescent protein), and sgRNA co-expression vector for efficient targeted disruption [28].

To analyze the disruption rate of the sgRNAs combined with Cas9 when targeting the *BCR* or *cABL* genomic loci, a T7 endonuclease I (T7E1) assay was employed as described previously [28]. In brief, after the Cas9-GFP/sgRNA vectors were transfected into K562 cells, genomic DNA was extracted and used as a template for PCR amplification of the fragments harboring the target sites. The PCR products were then denatured, reannealed, and digested using T7E1 (New England BioLabs, Inc., Ipswich, USA). Finally, the digestion products were analyzed via agarose gel electrophoresis, and the cleaved fragments were observed using Gel Imager software. The disruption rate was calculated as described previously [ 28, 29].

### Cas9-GFP/dual-sgRNA co-expression plasmid constructs

To construct a lentiviral plasmid co-expressing Cas9, GFP, and dual-sgRNAs, a sequence comprising hU6-sgRNA1-mU6-sgRNA2 ( Supplementary Table S1) was first synthesized with restriction sites for *Eco*RI and *Kpn*I at the 5′ and 3′ ends, respectively. The fragment was cloned and inserted into the vector GV518 (MCS-EF1a-Cas9-FLAG-P2A-EGFP; Genechem, Shanghai, China) and transformed into *Escherichia coli* competent cells. Colonies were then picked for sequencing. The verified recombinant vectors were subsequently used to produce lentiviruses.

### Lentivirus generation and transduction

Recombinant lentiviruses were produced by plasmid transfection of HEK293T cells [30]. Briefly, cells were seeded at 4×10 ^6^ cells/dish in 10-cm dishes the day before transfection. The recombinant lentiviral plasmids including Cas9-GFP/dual-BCR-sgRNA (containing *BCR* sgRNA1 and *BCR* sgRNA2), Cas9-GFP/dual-BA-sgRNA (containing *BCR* sgRNA1 and *cABL* sgRNA1), Cas9-GFP/hU6-sgRNA (BCR exon1-1), Cas9-GFP/hU6-sgRNA (BCR exon1-2), and Cas9-GFP/hU6-sgRNA1 (BCR exon1-1)-mU6-sgRNA2 (BCR exon7), together with the helper plasmids pSPAX2 and pMD2G (plasmid ratio 4:3:1) were cotransfected into HEK293T cells to package lentivirus using Lipofectamine 2000 (Invitrogen, Waltham, USA). The control empty vector Cas9 was co-transfected into HEK293T cells together with the auxiliary plasmids pSPAX2 and pMD2G to produce the virus, as the negative control virus (NC). The medium was collected after 48, 72, and 96 h, cleared by low-speed centrifugation, and filtered through 0.45-μm polyvinylidene fluoride (PVDF) filters (Millipore, Billerica, USA). A universal virus concentration kit (Beyotime Biotechnology, Shanghai, China) was used to concentrate the viral stocks. Viral aliquots were stored at –80°C.

K562 and KBM5 cells were seeded into 6-well plates at approximately 1‒2×10 ^6^ cells per well, 200 μL of concentrated virus was added, and polybrene (a polymeric quaternary amine) was added at a final concentration of 5 μg/mL to the medium. Cells were reinfected 24 h later, and at 24 h after the second infection, the cells were cultured in medium supplemented with 10% FBS for 3 days. Fluorescence was then observed under an inverted fluorescence microscope.

### Selection of Cas9/dual-sgRNA-mediated targeted disruption of *BCR-ABL* fusion gene clones

K562 and KBM5 cells infected with Cas9/dual-sgRNA lentivirus were collected, harvested, and rinsed with 1× phosphate-buffered saline (PBS). GFP-positive cells were isolated using fluorescence-activated cell sorting (FACS) on a flow cytometer (FACS Aria; BD Biosciences, San Jose, USA), plated as single cells in 96-well plates, and incubated at 37ºC for two weeks. The selected clones were expanded for subsequent experiments.

### Cell proliferation assay

Cell proliferation was measured by cell counting kit 8 (CCK-8) assay. The densities of K562 cells, KBM5 cells, and selected clone cells were counted, the cells were diluted to 5×10 ^4^ cells/mL, and 5000 cells were seeded in 96-well plates and set with six replicate wells (a medium blank control group was set up each time). CCK8 reagent (Beyotime Biotechnology) was added at 0, 24, 48, and 72 h (20 μL/well), and the plates were incubated at 37°C for 2 h. The plates were subsequently placed on a microplate reader (Varioskan Flash; Thermo Fisher Scientific, Waltham, USA), after which the absorbance at 450 nm was read. The optical density (OD) of the cells was calculated at each time point, cell growth curves were plotted, and the growth rates of the selected clones were compared with those of the control cells (normal cells not transfected with plasmids or without virus infection). The experiments were repeated three times.

### Primary cells

The collection of samples from patients with CML (BCR-ABL P210) was reviewed and approved by the Institutional Review Board (the Ethical Committee of the First People’s Hospital of Chenzhou), and all patients provided informed consent. Briefly, CD34 ^+^ stem/progenitor cells were separated from the bone marrow (BM) or peripheral blood mononuclear cells of five patients with t(9;22)-positive CML (CML-CP) and one ALL donor (CML-BP) with the P210 *BCR-ABL* fusion gene using positive immunomagnetic column separation (Miltenyi Biotec, Inc., Auburn, USA) as described previously ( Supplementary Table S2) [31]. Briefly, BM mononuclear cells (BMMNCs) were isolated using Ficoll density gradient centrifugation, and CD34 ^+^ cells were selected from BMMNCs using positive immunomagnetic column separation (Miltenyi Biotec) according to the manufacturer’s instructions. The purity of the cells ranged from 80% to 95%, as determined by flow cytometry, and their viability was above 90%, as detected by trypan blue exclusion assay.

Primary CD34 ^+^ leukemic cells isolated from patients with CML were cultured as described previously [26]. Briefly, CD34 ^+^ cells were cultured in 6-well plates in medium without serum but supplemented with growth factors including 100 ng/L granulocyte-macrophage colony-stimulating factor (GM-CSF), 500 ng/L granulocyte colony stimulating factor (G-CSF), 100 ng/L stem cell factor (SCF), 25 ng/L leukemia inhibitory factor (LIF), 100 ng/L macrophage inflammatory protein 1-alpha (MIP-1α), and 1 μg/L interleukin 6 (IL-6). All cytokines were purchased from Stem Cell Technologies (Vancouver, Canada).

### Clonogenic assay

K562, KBM5, and their derived cells were collected by centrifugation, rinsed twice with precooled PBS, and resuspended in complete medium. The cells were counted, and 500 or 1000 cells were seeded in 35-mm cell plates supplemented with 2 mL of methylcellulose semisolid medium H4230 (without cytokines; Stem Cell Technologies) supplemented with 10% FBS. The cell plates were placed in an incubator for 10–14 days, after which the cell colonies were counted and the percentage relative to the control cells was calculated. The experiments were repeated at least three times.

CD34 ^+^ stem/progenitor cells separated from patients with CML (BCR-ABL P210) or ALL (Ph+BCR-ABL P210) were infected with Cas9/dual-sgRNA lentivirus or control virus for 24 h, after which the cells were harvested, rinsed with 1× PBS, and used for clonogenic assay. Clonogenic assays were carried out using methylcellulose medium supplemented with the recombinant cytokine MethoCult H4434 [containing human interleukin 3 (IL-3), GM-CSF, SCF and erythropoietin; for human origin CD34 ^+^ cells] (Stem Cell Technologies) according to the manufacturer’s technical manual. The total colony-forming units (CFU-total) were counted as previously described [31]. Data were normalized to those of cells without virus infection.

### Viable cell counting and cell apoptosis assay

CD34 ^+^ cells from three patients with CML in the chronic phase and one patient in the blast phase (ALL) were infected with Cas9-GFP/dual-BA-sgRNA lentivirus or control virus (or without virus) for 24 h. Following further culture for 72 h, the viable cells were counted using the trypan blue exclusion assay at 0, 24, 48 and 72 h. The percentage of survived cells relative to that of the control cells was calculated, and the cell growth curve was determined using the viable cell percentage.

K562 and KBM5 cells were infected with Cas9-GFP/dual-BA-sgRNA lentivirus or control virus (or without virus) for 48 h, harvested, rinsed with 1× PBS, and subjected to cell apoptosis assay. Cell apoptosis was evaluated using an Annexin V (AV) detection system with an AV-FITC kit (fluorescein isothiocyanate; Clontech, BD Biosciences, Mountain View, USA) as previously described [31].

### Western blot analysis

Total protein was extracted from K562, KBM5, or CML primary cells using radioimmunoprecipitation assay (RIPA) buffer (Beyotime Biotechnology). All protein concentrations were measured using a bicinchoninic acid (BCA) protein assay kit (Beyotime Biotechnology). Approximately 40 μg of protein extract per sample was separated via 8% or 10% SDS-PAGE and subsequently transferred onto a PVDF membrane (Millipore). Next, 5% bovine serum albumin or 5% non-fat milk was used to block the membranes. The membranes were then incubated with anti-BCR, anti-cABL, antiphospho-p-cABL, anti-signal transducer and activator of transcription (STAT5), anti-p-STAT5, anti-p-mitogen activated kinase (MAPK), anti-p-extracellular regulated kinase (ERK), anti-β-catenin, and anti-cyclinD1 antibodies (Cell Signaling Technology, Danvers, USA); anti-c-myc, anti-poly(ADP-ribose) polymerase (PARP), anti-cleaved PARP, antipro-caspase3, and anti-cleaved caspase3 antibodies (Abcam, Cambridge, USA); and anti-β-actin (Sigma, St Louis, USA) overnight at 4°C, followed by incubation for 1 h with horseradish peroxidase (HRP)-conjugated secondary antibodies (Sigma). After extensive washing in Tris-buffered saline-Tween 20 (TBST), the levels of the immunoreactive proteins were detected using an enhanced chemiluminescence (ECL) kit (Cell Signaling Technology) followed by Quantity-One software analysis (Bio-Rad Laboratories, Hercules, USA). β-Actin was used as the loading control.

### CML mouse model

Nonobese diabetic/severe combined immunodeficiency (NOD/SCID) mice were purchased from Hunan SLAC Jingda Laboratory Animals Company (Changsha, China). KBM5 cells, KBM5-NC cells, or KBM5-Cas9-BA cells were injected via the tail vein (5×10 ^6^ cells per mouse) [32]. Four weeks after tail vein injection, GFP ^+^ cells in the peripheral blood of the mice in each group were counted. Two months after tail vein injection, two mice in each group were sacrificed, and their livers and spleens were collected and subjected to hematoxylin and eosin (HE) staining. All animal experiments were approved by the Ethical Committee of the First People’s Hospital of Chenzhou, University of South China. The Laboratory Animal Guidelines for Ethical Review of Animal Welfare of China were followed to ensure the welfare of the mice.

### Off-target assay

Genome-wide profiling of CRISPR-Cas9 off-target effects in Cas9/dual-BA-sgRNAs lentivirus infected K562 cells and control cells (untreated group, without virus infection) were analyzed using next-generation sequencing (NGS) or high-throughput sequencing of whole-genome DNA. Briefly, genomic DNA was digested *in vitro* and subjected to whole-genome sequencing (WGS) by BGI Genomics Co., Ltd. (Shenzhen, China). Offtarget effects were determine by both WGS data analysis and analysis using Cas-OFFinder [33]. This method is more sensitive and comprehensive than cell-based methods for identifying offtarget sites.

### Statistical analysis

SPSS 20 (IBM Corp., Armonk, USA) was used for the data analysis, and data are expressed as the mean ± the standard deviation (SD). Student’s *t* test and one-way analysis of variance (ANOVA) were applied to determine significant differences between two groups and among multiple groups, respectively. In these analyses, *P* values were two-sided, and *P*<0.05 was considered statistically significant.

## Results

### Dual-BCR sgRNAs and Cas9 efficiently disrupts the *BCR-ABL* fusion gene

To determine the p210 *BCR-ABL* fusion gene sequence, a pair of primers (BA-3.5 kb-F and BA3.5 kb-R) was designed to amplify the genomic DNA of CML cells. Agarose gel electrophoresis revealed a specific *BCR-ABL* PCR product of approximately 3.5 kb ( Supplementary Figure S1A), which was confirmed by Sanger sequencing ( Supplementary Figure S1B).

**Figure 1:**
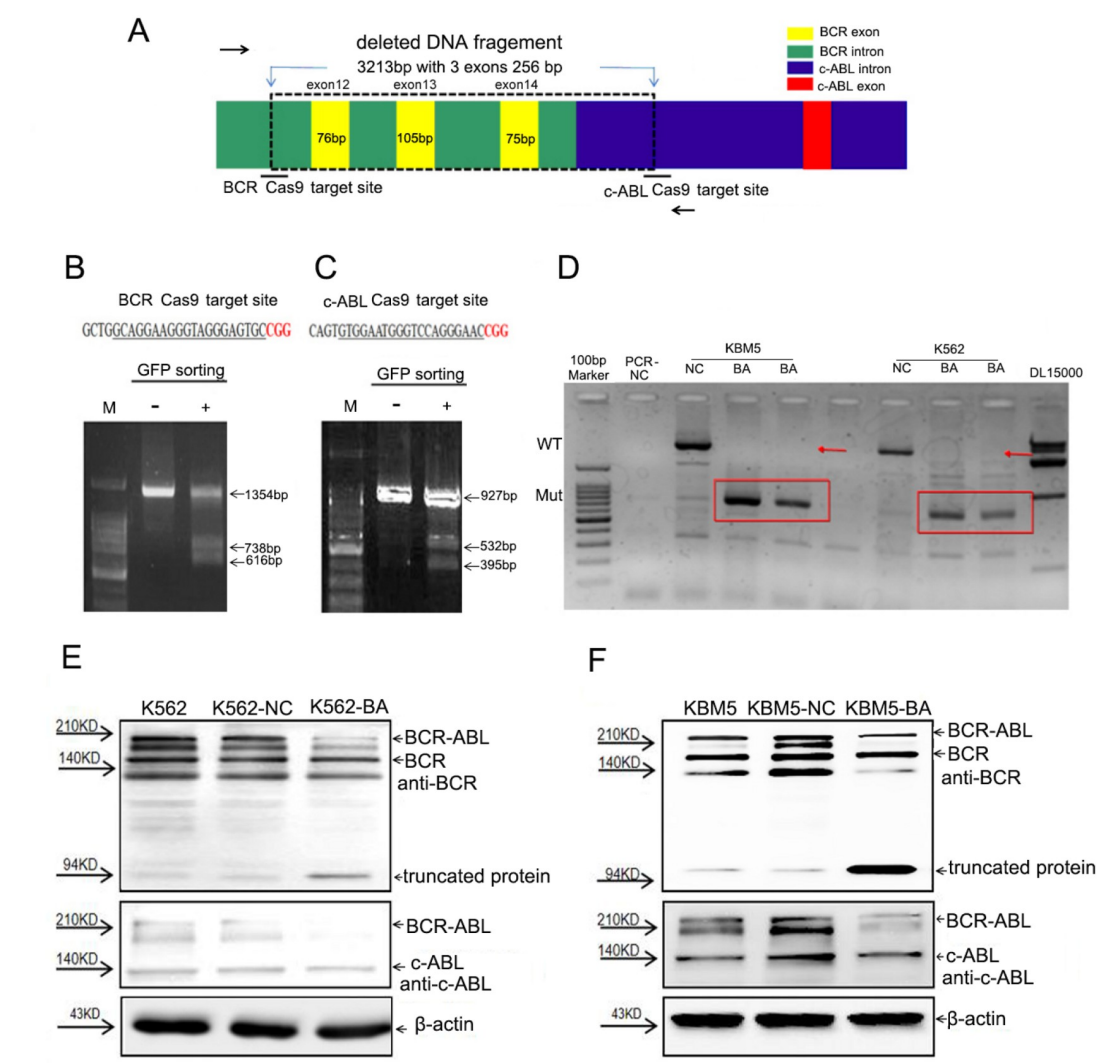
Figure 1 Targeted disruption of the *BCR-ABL* fusion gene by Cas9-GFP/dual-BA-sgRNA in CML cells (A) Schematic diagram of the targeted disruption of the BCR-ABL fusion gene using dual sgRNAs which target a BCR intron and a cABL intron, respectively. (B,C) Assay of the targeting efficiency of the two selected sgRNAs using the T7E1 method. The sgRNAs were cloned into MCS-pCS2-CMV-Cas9-IRES-GFP vector to construct a Cas9, GFP, and sgRNA co-expression vector, which was transfected into K562 cells and the sorted GFP+ cells were used for a targeted disruption efficiency assay. M, Marker, DNA molecular weight. (D) GFP+ cell clones derived from Cas9-GFP/dual-BA-sgRNA lentivirus infected K562 and KBM5 cells were used to analyze BCR-ABL fusion genomic disruption by PCR. The arrowheads indicate the wild-type fragments, and the frames indicate the mutation fragments. M, DNA marker; PCR-NC, PCR negative control. (E,F) The level of the BCR-ABL fusion protein in Cas9-GFP/dual-BA-sgRNA lentivirus infected K562 and KBM5 cells, as determined by western blot analysis. KBM5-NC&K562-NC, control virus infected CML cells; KBM5-BA&K562-BA, Cas9-GFP/dual-BA-sgRNA lentivirus infected K562 and KBM5 cells. Arrowheads indicate the BCR-ABL fusion protein (P210 kDa), BCR protein, BCR truncated protein (94 kDa), cABL protein, and β-actin, respectively. CML, chronic myeloid leukemia; BCR, breakpoint cluster region protein; ABL, Abl Oncogene 1, Receptor Tyrosine Kinase; GFP, green fluorescent protein; T7E1, T7 endonuclease I; Cas9, CRISPR-associated protein 9; sgRNA, single guide RNA.

To disrupt the genomic sequence of *BCR-ABL*, dual-sgRNA Cas9 was used to delete a DNA fragment containing the exon sequence ( Figure 1A and Supplementary Figure S2). Several sgRNA targeting sites in the introns of *BCR* and *cABL* were selected ( Supplementary Table S1). To evaluate the targeting efficiency of the single sgRNAs, two reverse complementary DNA oligonucleotides were synthesized, annealed, and cloned and inserted into the vector pCS2-CMV-Cas9-IRES-GFP to construct a Cas9, GFP, and sgRNA co-expression vector. The targeted disruption efficiency of these sgRNAs was assayed using T7E1 after the Cas9-GFP/sgRNA vectors were transfected into K562 cells, and GFP ^+^ cells were sorted by FACS ( Figure 1B,C and Supplementary Figure S3).

To verify the efficiency of using Cas9 and dual sgRNAs for the targeted disruption of *BCR-ABL*, two sgRNAs targeting a *BCR* intron combined with two *U6* promoters were synthesized and cloned and inserted into the lentiviral vector GV518. The virus was used to infect K562 cells, and the GFP ^+^ cells were sorted via FACS. The GFP ^+^ cells were cultivated and expanded to detect the targeted disruption efficiency at the genomic DNA and protein expression levels ( Supplementary Figures S4‒ S6). Cas9 and the dual sgRNAs effectively disrupted the *BCR-ABL* fusion genomic sequence and affected protein expression. As expected, this strategy resulted in a frameshift mutation in *BCR-ABL*, producing a truncated BCR protein of approximately 94 kDa ( Supplementary Figure S6). However, in addition to the *BCRABL* fusion gene, *BCR* dual sgRNAs also guided Cas9 to cut the *BCR* gene ( Supplementary Figure S6).

### Efficiency of targeting the *BCR-ABL* genomic sequence using Cas9 and dual sgRNAs targeting *BCR* and *cABL* introns

Although it is common to use one or two sgRNAs to target the first exon of a gene’s open reading frame (ORF) to induce a frameshift mutation, this strategy is not suitable for knocking out fusion genes because these sgRNAs not only target the fusion genes but also directly target the constituent genes of the fusion genes, resulting in off-target effects. First, we used a single sgRNA to target the first exon of the *BCR* to disrupt *BCR-ABL*, and the results showed that after GFP ^+^ cell sorting, the expression levels of the BCR-ABL and BCR proteins decreased moderately in K562 and KBM5 cells ( Supplementary Figure S7). To obtain cells with complete disruption of the fusion gene, single-cell-derived cell clones must be screened out [ 34, 35]. Furthermore, the length of the DNA sequences targeted using double sgRNAs should not be too long; otherwise, the efficiency will be too low to obtain complete gene knockouts ( Supplementary Figure S8). Although the maximum length of a DNA fragment deleted successfully to date using the Cas9/sgRNA system was above 50 kb, the efficiency was very low; thus, reducing the fragment length would facilitate efficient editing [ 36- 38]. Herein, we chose to eliminate a 2000‒3000 bp DNA fragment of *BCR-ABL* using double sgRNAs to ensure high efficiency.

Therefore, to avoid off-target effects on genomic DNA as much as possible, dual sgRNAs that targeted *BCR* and *cABL* introns were used to guide Cas9 to target *BCR-ABLs* ( Figure 1A–C). After K562 and KBM5 leukemia cells were infected with Cas9-GFP/hU6-sgRNA1(BCR1)-mU6-sgRNA2 (cABL1) (Cas9-GFP/dual-BA-sgRNA) viruses, the GFP ^+^ cells were sorted via FACS and used for a targeting efficiency assay. The PCR results indicated that the *BCR-ABL* fusion gene was completely eliminated in the screened GFP ^+^ cells (named K562-BA and KBM5-BA, respectively), in which the 3.9 kb wild-type DNA fragment was absent and only the expected fragments appeared ( Figure 1D). Moreover, compared with those in control cells, the protein levels of BCR-ABL, as determined by western blot analysis, were significantly lower in K562-BA and KBM5-BA cells ( Figure 1E,F). This strategy resulted in a frameshift mutation in *BCR*- *ABL* that produced a truncated BCR protein of approximately 94 kDa ( Figure 1E,F). Intriguingly, the levels of the cellular BCR and cABL proteins were hardly affected ( Figure 1E,F).

### Inhibition of BCR-ABL tyrosine kinase signaling through Cas9-GFP/dual-BA-sgRNA-mediated disruption of the fusion gene in leukemia cells

**Figure 2:**
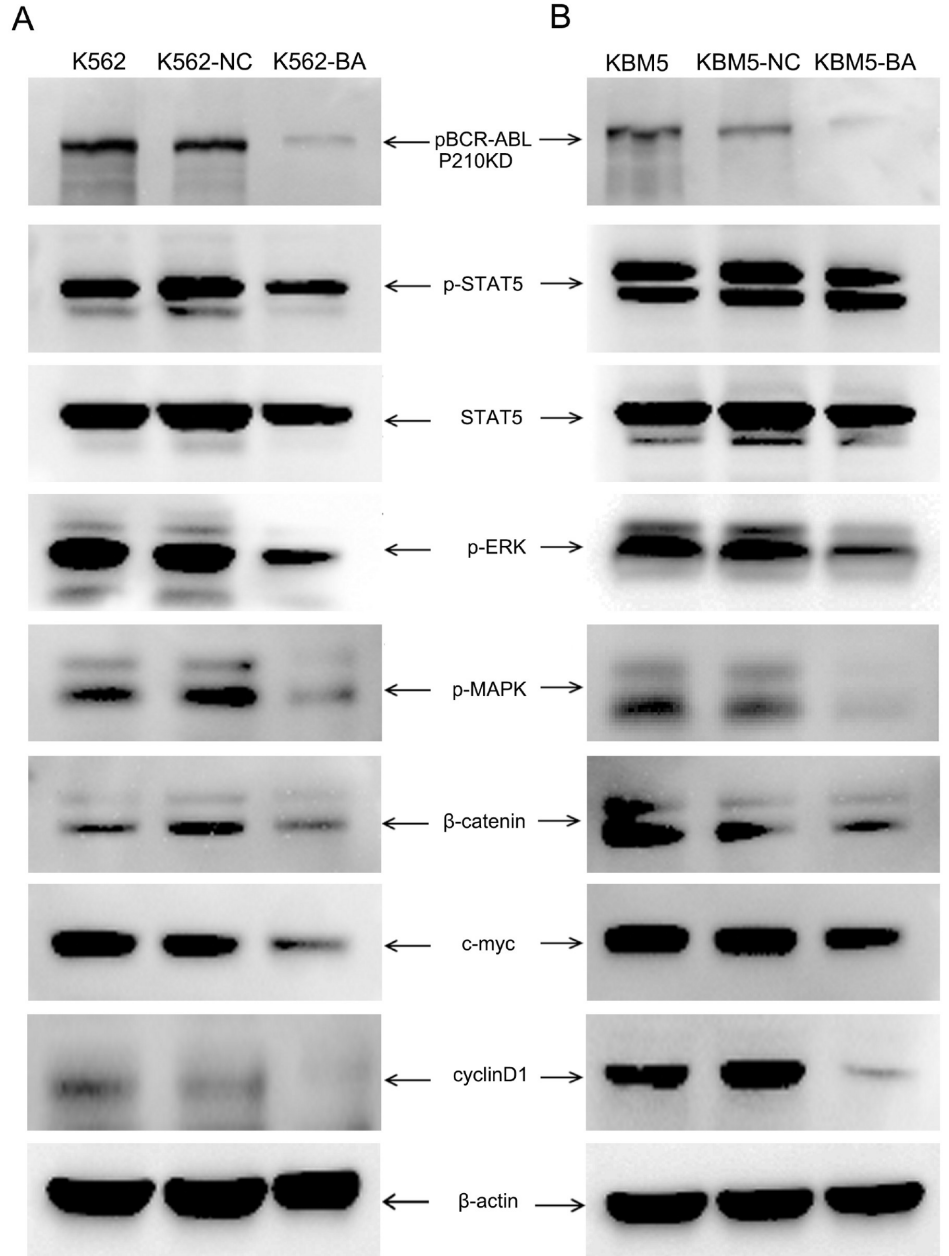
Figure 2 Inhibition of *BCR-ABL* tyrosine kinase signaling pathway and its downstream molecules through Cas9-GFP/dual-BA-sgRNA targeted disruption of fusion gene in leukemia cells GFP+ cell clones derived from Cas9-GFP/dual-BA-sgRNA lentivirus-infected K562 and KBM5 cells were cultivated and used to detect BCR-ABL phosphorylation and changes to its downstream molecules, (A) for K562 cells and (B) for KBM5 CML cells. KBM5-NC&K562-NC, control virus-infected CML cells; KBM5-BA&K562-BA, Cas9-GFP/dual-BA-sgRNA lentivirus-infected K562 and KBM5 cells. CML, chronic myeloid leukemia; BCR, breakpoint cluster region protein; ABL, Abl Oncogene 1, Receptor Tyrosine Kinase; GFP, green fluorescent protein; STAT5, signal transducer and activator of transcription; p-STAT5 phosphorylated STAT5; p-MAPK, phosphorylated mitogen activated protein kinase; p-ERK, phosphorylated extracellular regulated kinase; Cas9, CRISPR-associated protein 9; sgRNA, single guide RNA.

To investigate whether the Cas9-GFP/dual-BA-sgRNA system could block the BCR-ABL tyrosine kinase signaling pathway, the level of p-BCR-ABL and the changes in the levels of its downstream molecules, such as p-STAT5, p-MAPK, p-ERK, c-myc, β-catenin, and cyclin D1, were determined. After targeted knockdown of *BCR-ABL*, the level of p-BCR-ABL decreased significantly, and the levels of the above mentioned downstream signaling molecules were also downregulated in both K562-BA and KBM5-BA cells ( Figure 2A,B). The results were confirmed using single GFP ^+^-derived cell clones ( Supplementary Figure S9A,B). These results indicated that BCR-ABL tyrosine kinase signaling in CML cells was effectively inhibited by the targeted disruption of *BCR-ABL* by Cas9-GFP/dual-BA-sgRNA.

### Cas9-GFP/dual-BA-sgRNA inhibits cell proliferation and induces cell apoptosis in CML cells

**Figure 3:**
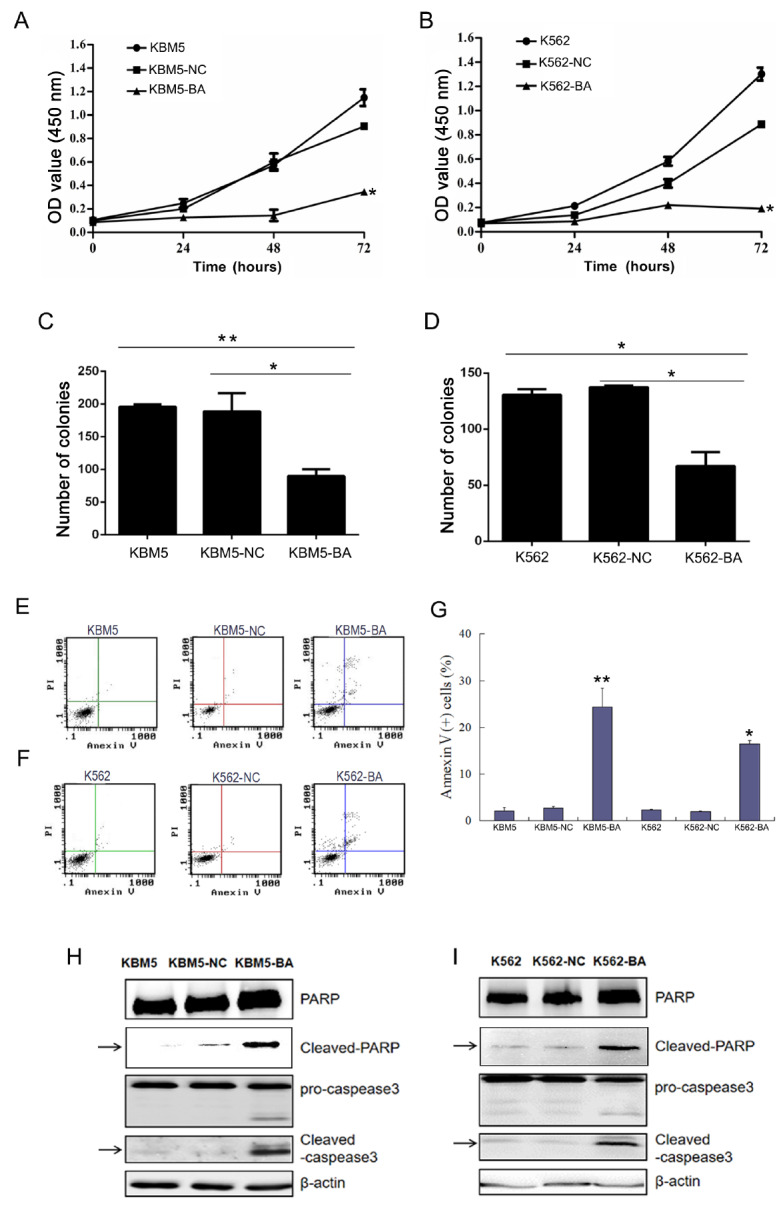
Figure 3 Inhibition of leukemia cell proliferation and induction of cell apoptosis by Cas9-GFP/dual-BA-sgRNA (A,B) GFP+ cells derived from Cas9-GFP/dual-BA-sgRNA lentivirus infected K562 and KBM5 cells were used for CCK-8 assays. (C,D) Cell clone formation experiments in methylcellulose semisolid medium H4230 (without cytokines). (E–G) Cell apoptosis was analyzed by FACS using and an AnnxinV kit. All experiments were repeated at least three times (*P<0.05, **P<0.01). (H,I) The expressions of apoptotic proteins were determined by western blot analysis. β-Actin was used as the loading control. KBM5-NC&K562-NC, control virus-infected CML cells; KBM5-BA&K562-BA, Cas9-GFP/dual-BA-sgRNA lentivirus-infected K562 and KBM5 cells; CML, chronic myeloid leukemia; GFP, green fluorescent protein; Cas9, CRISPR-associated protein 9; sgRNA, single guide RNA; CCK-8, cell counting kit 8; FACS, fluorescence activated cell sorting; PARP, poly(ADP-ribose) polymerase.

The effects of Cas9-GFP/dual-sgRNA on cell proliferation and apoptosis in K562-BA and KBM5-BA cells were analyzed by CCK-8 and clonogenic assays for proliferation and by analyzing Annexin V expression for apoptosis. The survival ability of K562-BA and KBM5-BA cells was significantly lower than that of the other groups, while there was no difference in proliferation between the control virus-infected cells (NC) and cells without virus infection (control) ( Figure 3A,B). Clonogenic assays revealed that the *BCR*- *ABL*-knockdown cells produced fewer colonies than the control cells ( Figure 3C,D and Supplementary Figure S9C,D). These data indicated that the proliferation of KBM5-BA and K562-BA cells was significantly inhibited.

Furthermore, Annexin V/FACS analysis revealed significantly greater apoptosis in KBM5-BA and K562-BA cells than in control cells ( Figure 3E–G). The levels of apoptotic proteins were also determined by western blot analysis, which demonstrated significantly increased activation of proapoptotic proteins in K562-BA and KBM5-BA cells ( Figure 3H,I). These results confirmed that Cas9-GFP/dual-BA-sgRNA induced apoptosis in CML cells.

### Prolonged lifespan of CML mice inoculated via the tail vein with Cas9-GFP/dual-BA-sgRNA lentivirus-infected leukemia cells (KBM5-Cas9-BA)

**Figure 4:**
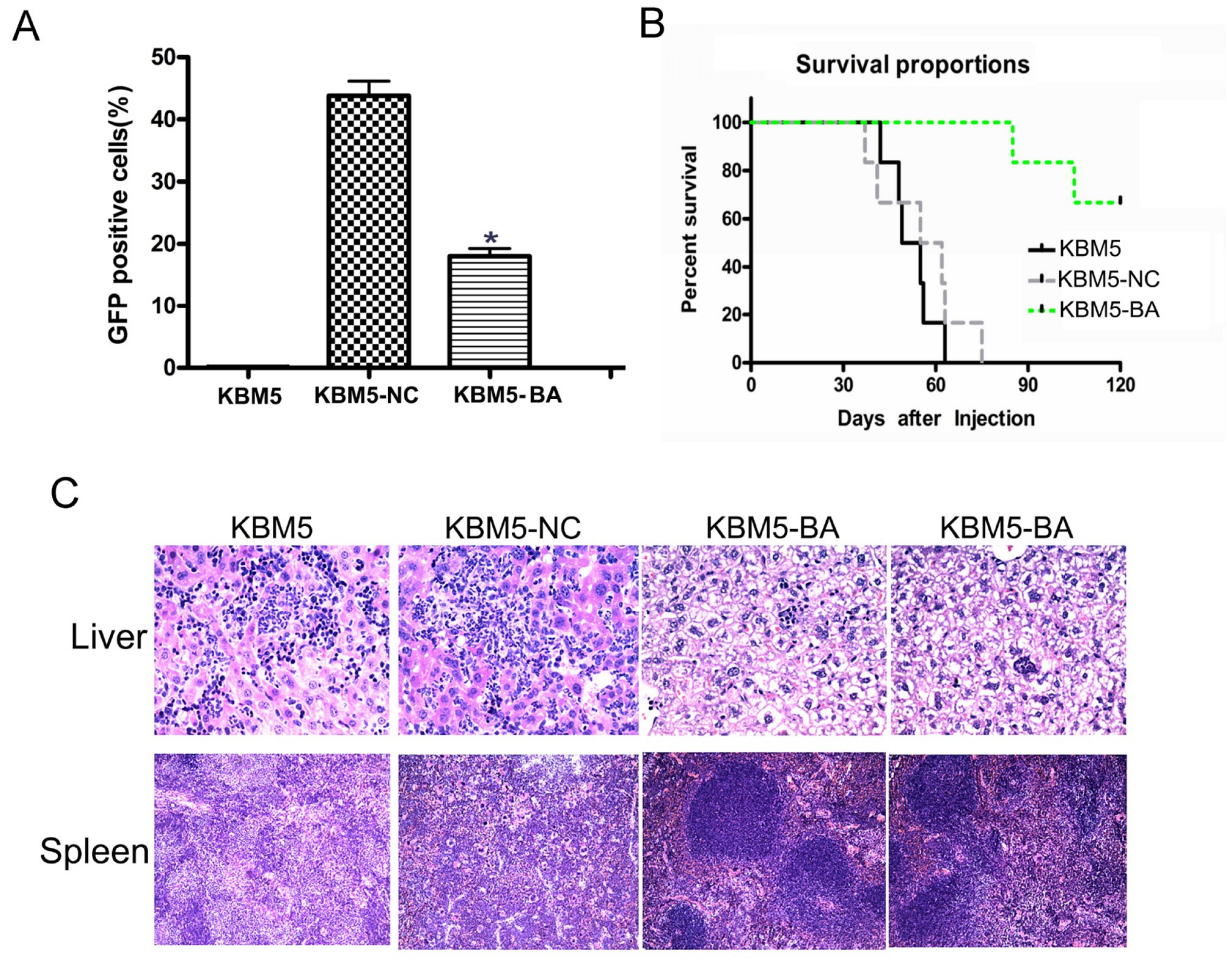
Figure 4 Cas9-GFP/dual-BA-sgRNA lentivirus prolonged the lifespan of CML mice model constructed by injection of leukemia cells via tail vein To construct the CML mice model, Cas9-GFP/dual-BA-sgRNA lentivirus-infected KBM5 cells (KBM5-Cas9-BA) were injected into NOD-SCID mice via the tail vein. (A) The percentage of GFP+ cells in the peripheral blood of CML mice was determined by FACS 4 weeks after cell injection. (B) Survival assay of CML mice after cell injection. (C) Cell infiltration in CML mice liver (×20) and spleen tissue (×10) were analyzed by HE staining (*P<0.05). KBM5-NC, control virus-infected cells; KBM5-BA, Cas9-GFP/dual-BA-sgRNA lentivirus-infected cells; CML, chronic myeloid leukemia; GFP, green fluorescent protein; Cas9, CRISPR-associated protein 9; sgRNA, single guide RNA; NOD-SCID, nonobese diabetic/severe combined immunodeficiency; FACS, fluorescence activated cell sorting; HE, hematoxylin and eosin.

To construct the CML mouse model, KBM5 cells and virus-infected cells were injected into NOD-SCID mice via the tail vein [32]. Four weeks after the injection of KBM5, KBM5-NC or KBM5-Cas9-BA cells, GFP ^+^ cells in the peripheral blood of each group were counted via FACS. The results showed that the percentage of GFP ^+^ cells in the KBM5-Cas9-BA group was significantly lower than that in the KBM5-NC group ( Figure 4A). In addition, the survival rate of mice injected with KBM5-Cas9-BA cells was markedly greater than that of mice injected with KBM5 and KBM5-NC cells ( Figure 4B). Two months after tail vein injection, two mice in each group were sacrificed, and their livers and spleens were subjected to HE staining to observe leukemia cell infiltration. The results indicated that the KBM5-Cas9-BA group had markedly lower leukemia cell infiltration in both the liver and spleen than the KBM5 and KBM5-NC groups ( Figure 4C). These results demonstrated that the Cas9-GFP/dual-BA-sgRNA lentivirus could prolong the lifespan of CML mice generated by injecting leukemia cells via the tail vein.

### Effects on primary cells of CML patients and on *BCR-ABL* fusion gene expression after infection with Cas9-GFP/dual-BA-sgRNA lentivirus

**Figure 5:**
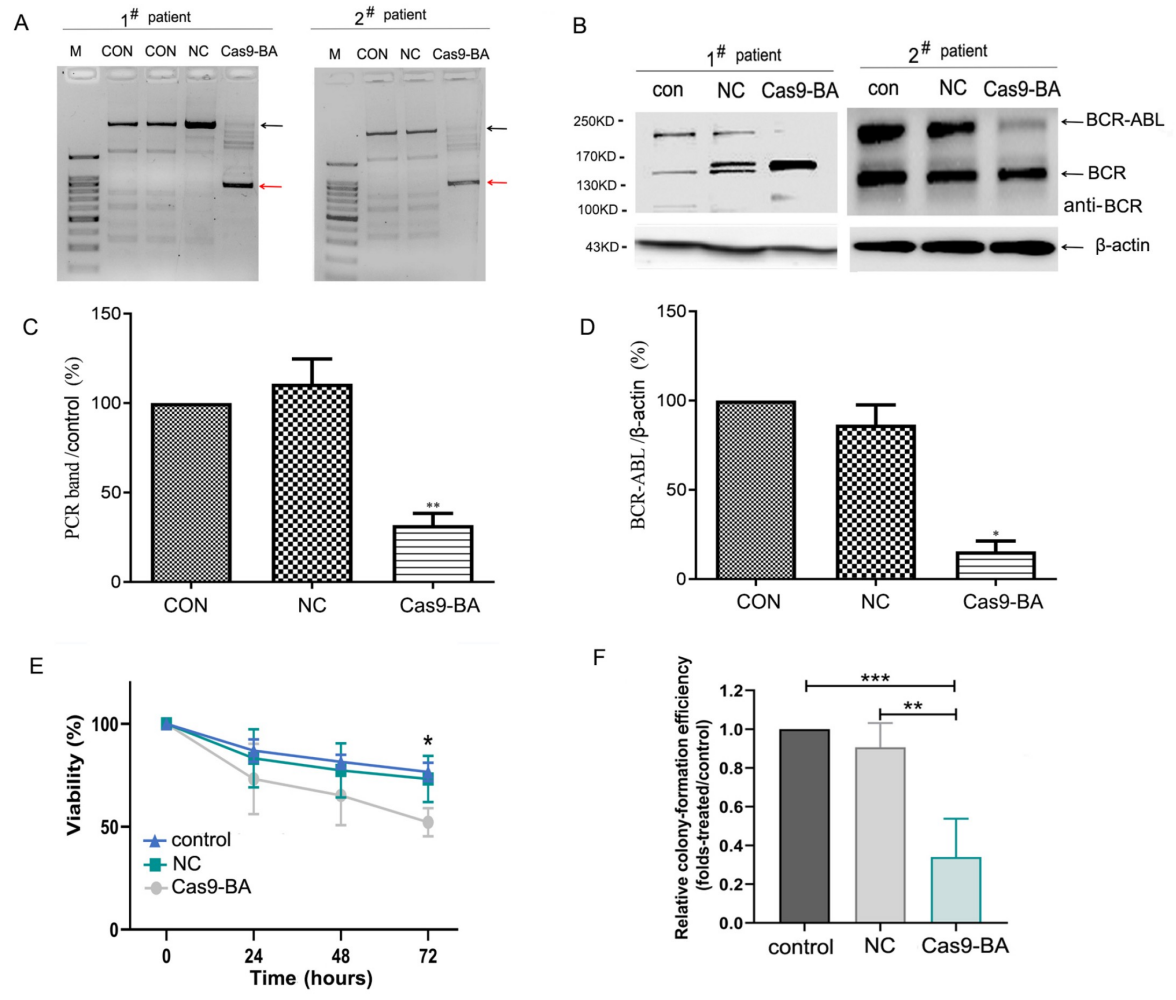
Figure 5 Effect of Cas9-GFP/dual-BA-sgRNA lentivirus infection on primary cells of CML patients and *BCR-ABL* fusion gene expression After CD34+ progenitor cells from patients with CML were infected with Cas9-GFP/dual-BA-sgRNA lentivirus, GFP+ cells were sorted and used to analyze the changes in the BCR-ABL genomic sequence, gene expression, cell survival and cell clone formation. (A) The disruption effect of the fusion gene genomic sequence in lentivirus-infected primary cells from patients with CML, as detected by PCR. The arrows above indicate the wild-type PCR fragments. The arrows below indicate the mutation type PCR fragments. Representative images of two patients are shown in the panel. (B) BCR-ABL fusion protein levels in lentivirus infected primary cells from patients with CML, as detected by western blot analysis using anti-BCR antibodies. Representative images of two patients are shown in the panel. For quantitation of PCR (C) and western blot bands (D), the bands of four patients with CML were quantified using Image J software, and the relative expression level of BCR-ABL was calculated. (E) Cell survival assay of CML primary cells using trypan blue exclusion after virus infection. (F) Clone forming assay of lentivirus-infected primary cells using methylcellulose medium with recombinant cytokines. Control, primary cells without virus infection; NC, primary cells infected with control virus; Cas9-BA, primary cells infected with Cas9-GFP/dual-BA-sgRNA lentivirus. The primary cells of all six patients with CML were used for cell survival and clone forming assays, and that of four patients with CML were used for analysis of the changes in the fusion gene genomic sequence and gene expression. *P<0.05, **P<0.01, ***P<0.001. CML, chronic myeloid leukemia; GFP, green fluorescent protein; Cas9, CRISPR-associated protein 9; sgRNA, single guide RNA; BCR, breakpoint cluster region protein; ABL, Abl Oncogene 1, Receptor Tyrosine Kinase.

The effect of the Cas9-GFP/dual-BA-sgRNA lentivirus on primary cells derived from patients with CML was investigated. CD34 ^+^ stem/progenitor cells isolated from five patients with CML-CP and one with CML-BP (ALL) ( Supplementary Table S2) were infected with Cas9GFP/dualBA-sgRNA lentivirus (Cas9-BA) or control virus (NC) or without virus, and then, the GFP ^+^ cells were sorted and used to analyze the genomic sequence alterations, fusion protein levels, cell survival, and cell colony formation. According to the results shown in Figure 5A,C, after infection with the Cas9-GFP/dual-BA-sgRNA lentivirus, the BCR-ABL fusion genomic sequence in the primary CML cells was almost completely disrupted; after PCR amplification, the longer DNA fragment (wild-type) disappeared, and only the mutated fragments appeared. Western blot analysis revealed that the level of the BCR-ABL fusion protein was markedly lower in Cas9-GFP/dual-BA-sgRNA lentivirus-infected cells (Cas9-BA) than in control cells and NC virus-infected cells, indicating that fusion gene expression was disrupted ( Figure 5B,D). A postinfection cell survival assay showed that the percentage of viable cells in the Cas9-BA group was markedly lower than that in the control group and NC group at 48 and 72 h ( Figure 5E). Moreover, after infection, the clonogenic ability of Cas9-BA-infected primary cells was also significantly lower than that of control and NC cells ( Figure 5F).

### The results of off-target assay in K562-Cas9-BA cells (cells infected by dual-BA-sgRNA lentivirus)

**Figure 6:**
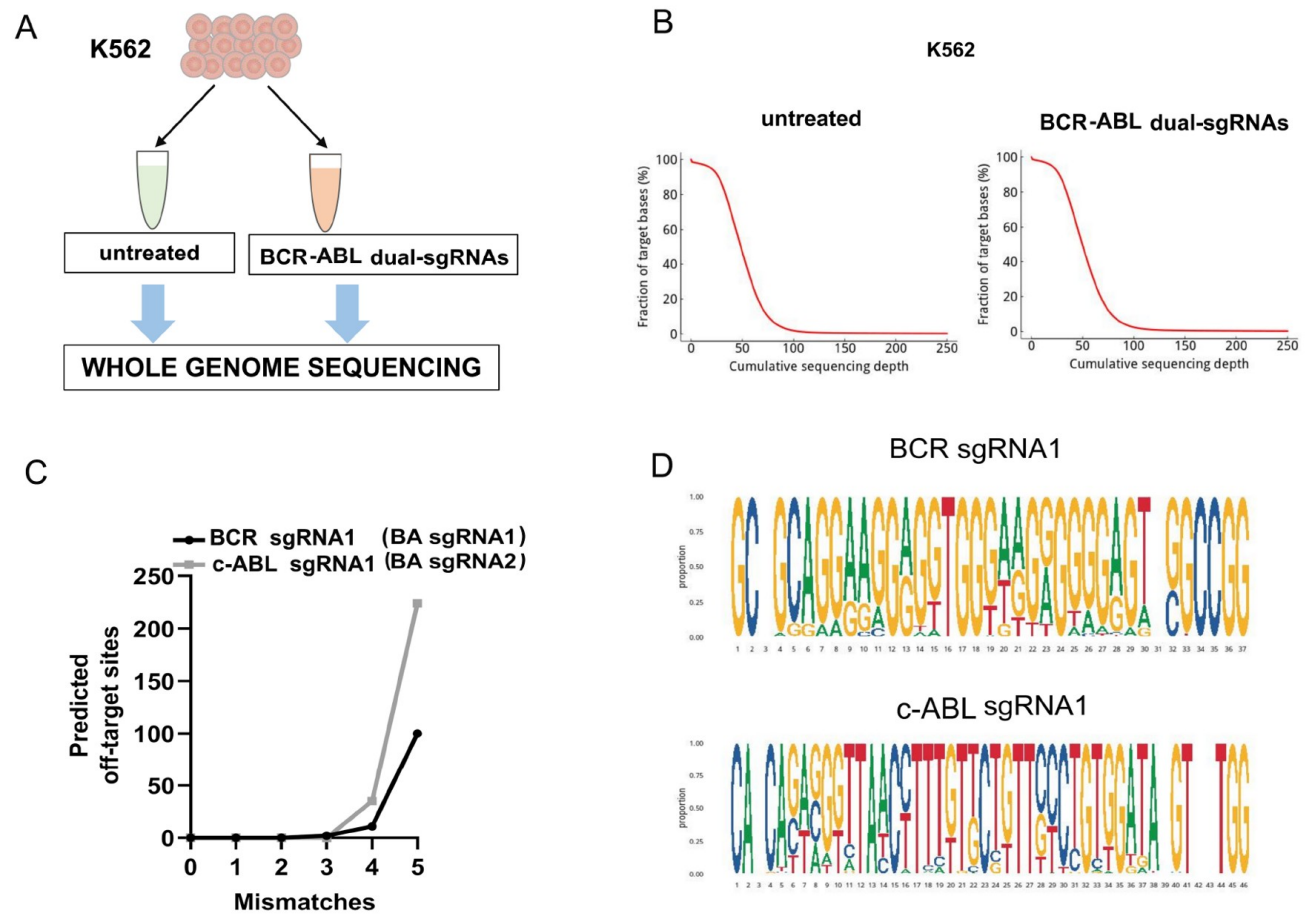
Figure 6 Off-target assay in K562-Cas9-BA (dual-BA-sgRNA) cells (A) Samples were subjected to whole-genome sequencing (WGS). (B) Cumulative sequencing depth distribution. The X-axis represents the sequencing depth and the Y-axis represents the proportion of the target area that reaches the corresponding sequencing depth and above. (C) Numbers of Cas-OFFinder-predicted gRNA-dependent off-target sites with up to 5 mismatches to the target sequence. (D) Base composition of the potential off-target site. The horizontal coordinates are the base positions of the homologous region sequences, and the proportions represent the composition of the corresponding bases. Cas9, CRISPR-associated protein 9; sgRNA, single guide RNA; gDNA, genomic DNA.

CRISPR/Cas9-induced off-target events were investigated by whole-genome sequencing (WGS) ( Figure 6A), and insertion‒deletion (InDel) mutations were evaluated in predicted sgRNA homologous regions and at the genome-wide level. Our WGS produced a stable overall depth of genome coverage for K562 untreated cells and K562 BCR-ABL dual-sgRNA-treated cells (51.3× the mean of the untreated samples and 52.6× the mean for the BCR-ABL dual-sgRNA samples; Figure 6B).

Cas-OFFinder *in silico* analysis combined with WGS data was used to predict potential off-target sites. There were 113 off-target sites for *BCR-ABL* sgRNA1 (BCR sgRNA1) and 259 off-target sites for *BCR-ABL* sgRNA2 (cABL sgRNA1), allowing mismatches between the gRNA and target sequences ( Supplementary Tables S3 and S4). Notably, no predicted off-target sites allowing for one or two mismatches between the gRNA and target sequences were predicted for BCR sgRNA1 or cABL sgRNA1. Specifically, for BCR sgRNA1, there were 2 off-target sites allowing 3 mismatches, 11 off-target sites allowing 4 mismatches, and 100 off-target sites allowing 5 mismatches. For BCR sgRNA2, there were 35 off-target sites, allowing 4 mismatches, and 224 off-target sites, allowing 5 mismatches ( Figure 6C). Subsequently, we analyzed the sequences of the potential off-target sites and provided the base composition of the sequences of the sgRNA homology regions where the potential off-target sites are located ( Figure 6D).

Taken together, our results demonstrated that Cas9/dual-sgRNA could efficiently target and disrupt *BCR-ABL* at the genomic level. This led to gene expression downregulation and suppression of the BCR-ABL tyrosine kinase signaling pathway to induce CML cell apoptosis and inhibit proliferation *in vitro* and *in vivo*, suggesting a potential application for CML gene therapy.

## Discussion

Although anti-leukaemia drugs, such as TKIs, have been successfully applied for CML therapy, the treatment efficacy for patients with CML is limited because of drug resistance or disease relapse after therapy. One of the reasons is that the inhibitors target only the fusion protein to reduce kinase activity rather than the genomic sequence of the fusion gene and thus cannot eliminate the oncogene. The development of novel therapeutic strategies for CML is essential and urgently needed to improve the lifespan of patients with CML. Using CRISPR/Cas9 gene editing technology to disrupt the fusion gene at the genomic level would theoretically eliminate the abnormal fusion protein completely, representing a potential strategy for CML gene therapy.

Genome editing was optimized to increase on-target efficiency and reduce off-target efficiency [39]. The CRISPR/Cas9-GFP/dual-BA-sgRNA system is an evolutionary version based on CRISPR/Cas9. The success of this approach strongly depends on the presence of paired dual-sgRNAs and the presence of a protospacer adjacent motif (PAM), which markedly improves the specificity of the system and reduces the off-target effects of CRISPR/Cas9/dual-sgRNA. In our study, we designed several pairs of sgRNAs with different spacers and then selected the most efficient pair to target *BCR-ABL*. *In vitro*, we investigated the ability of CRISPR/Cas9/dual-sgRNA to disrupt the *BCR-ABL* oncogene and its effect on CML cell lines and primary cells isolated from patients with CML and explored its effect in a xenograft CML model. WGS analysis indicated that 116 and 261 offtarget effects (inDels) were caused by the two sgRNAs in K562 cells in our study ( Figure 6 and Supplementary Tables S3 and S4). These data showed that the developed method did not cause wide-ranging off-target effects, and the numbers of offtarget effects were comparable to those reported previously [ 23, 35, 40]. The balance between off-target effects and the efficiency of gene knockout is one of the main concerns when using CRISPR technology. Fortunately, the current online sgRNA design tools for predicting off-target effects are more accurate than previous versions, helping us to choose the optimal sgRNAs for each target gene sequence. Although it is impossible to completely avoid off-target effects, it is clear from our data that designing sgRNAs to target the intron sequences of the fusion genes separately could significantly prevent direct targeting of the two constituent gene sequences of the fusion gene and might be an effective strategy for knocking out or disrupting other fusion genes.

We also recommend that a fluorescent tag (GFP) be inserted into the CRISPR/Cas9/dual-sgRNA system for more intuitive observation. The developed method could be used to effectively delete target sequences to disrupt the expression of *BCR-ABL* without disrupting the expressions of normal *BCR* and *ABL* genes by targeting the CRISPR/Cas9 apparatus to two introns, one each in *BCR* and *cABL*, leading to the appearance of a truncated BCR-ABL protein. A recent study described a method in which two introns flank all broken regions [26], which is somewhat similar to our method. However, the deleted fragment in our system was shorter (comprising only *BCR* exons 12, 13, and 14 and *ABL* exon 1) and had less impact on the deleted cells. Moreover, our approach does not affect the sequences of the exons or the expression of the wild-type alleles involved in the rearrangement. These improvements are worthy of exploration for further clinical application. Despite the efficiency of targeted destruction guided by double sgRNAs, although we detected complete knockout of the fusion gene by PCR and the level of the fusion protein was markedly decreased, bands representing the full-length fusion protein still remained in each assay. This phenomenon has been reported in previous research [ 21, 23, 26]; thus, the underlying mechanism should be determined.

Although multiple clinical trials using CRISPR/Cas9 editing technology are ongoing worldwide, there are many problems to be addressed in the use of large-scale gene editing technology. The development of safe and effective methods for *in vivo* delivery remains the biggest challenge for the widespread clinical use of CRISPR/Cas9 in human therapy. Most current clinical studies use viral vectors; however, challenges such as immunogenicity, cytotoxicity and carcinogenicity still need to be overcome [41]. A recent report provided a more specific and safer therapy for CML by combining the CRISPR/Cas9 system with a nanoparticle delivery system, representing a good carrier for the *in vivo* delivery of CRISPR/Cas9 plasmids by optimizing the formulation of poly(ethylene glycol)-b-poly(lactic acid-co-glycolic acid) (PEG-PLGA)-based cationic lipid-assisted polymeric nanoparticles (CLANs). With the optimized formulation, CLANs were able to efficiently encapsulate pCas9 and protect pCas9 from DNases in physiological environments. In addition, CLAN was able to deliver CRISPR/Cas9 plasmids into CML cells both *in vitro* and *in vivo* [42]. However, sustained-release systems using nanocarriers still need to overcome many physical obstacles to realize CRISPR/Cas9 editing at the tumor site and implement precise treatment [39]. Moreover, advances in biomaterials will help expand the medical applications of genome editing in the future.

In summary, we showed that CRISPR/Cas9-GFP/dual-BA-sgRNA can efficiently edit *BCR-ABLs* with high specificity and flexibility. Despite the many therapeutic strategies being explored for treating patients with CML in recent decades, eliminating CML is challenging because of the emergence of TKI resistance and disease relapse. In this study, we established a virus-mediated Cas9/dual-sgRNA-mediated disruption of the BCR-ABL fusion gene that could significantly reduce Ph expression and abolish leukemia cell survival and tumorigenic abilities *in vitro* and *ex vivo*. The results suggested that this CRISPR/Cas9-based gene therapy has strong potential and might be further applied to treat patients with CML who are insensitive or resistant to imatinib treatment. We believe that the CRISPR/Cas9-GFP/dual-BA-sgRNA system developed in this study is a promising therapeutic option for patients with CML with TKI resistance or disease relapse.

## Supporting information

23362Supplementary_Data-20231128

## Supplementary Data

Supplementary data is available at *Acta Biochimica et Biophysica Sinica* online.
