# Supplementary material for: Targeted disruption of the BCR-ABL fusion gene by Cas9/dual-sgRNA inhibits proliferation and induces apoptosis in chronic myeloid leukemia cells : Cas9/Dual-sgRNA targeting of the BCR-ABL fusion gene
Source: Acta Biochim Biophys Sin (Shanghai). 2024 Feb 28;56(4):525–37. doi: 10.3724/abbs.2023280 (PMC11090847; doi:10.3724/abbs.2023280)
Supplement: 23362Supplementary_Data-20231128 [file 23362Supplementary_Data-20231128.pdf]

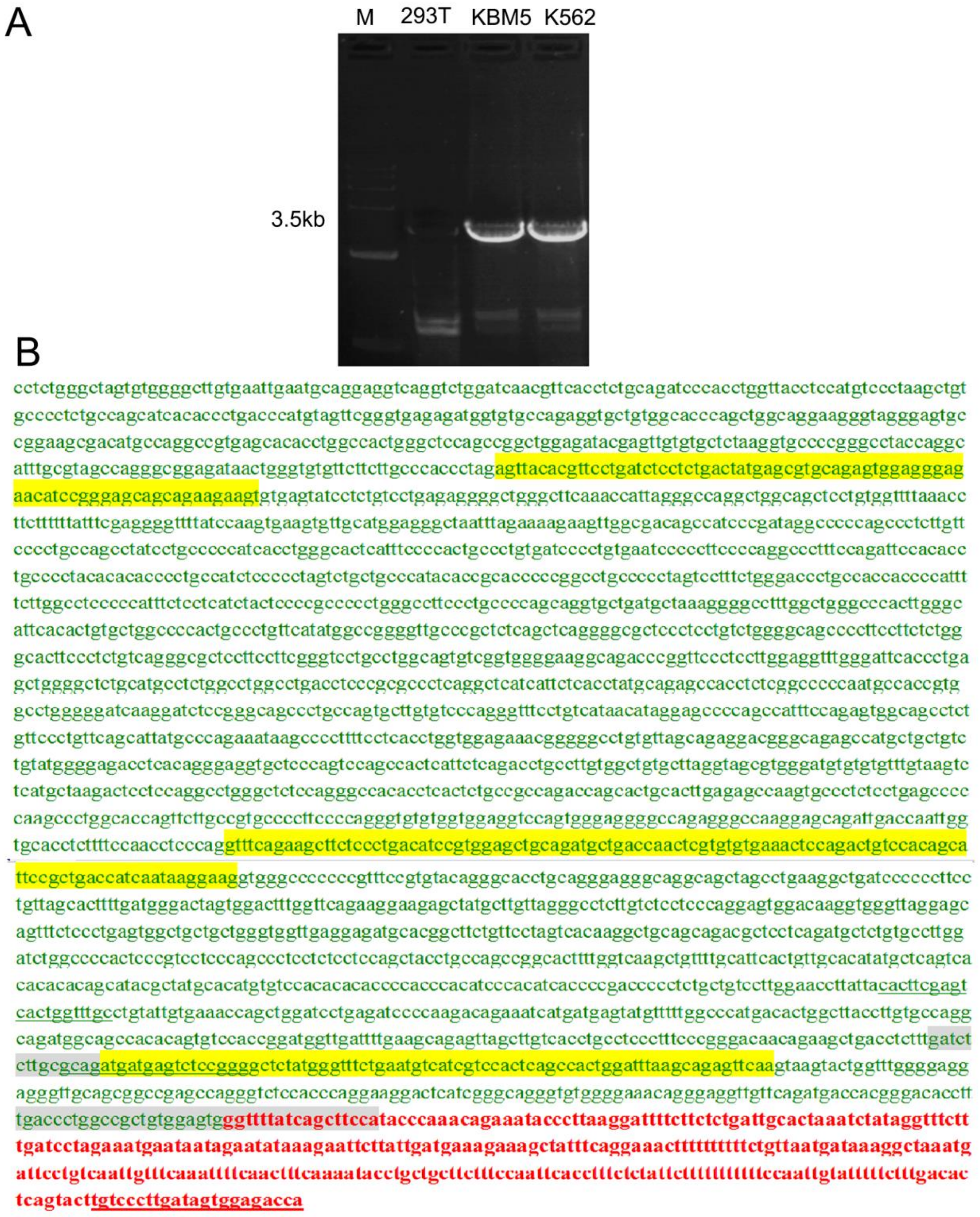

Supplementary Figure S1. Verification of the p210 BCR-ABL fusion genomic sequence

(A) PCR products amplified by primer pairs BA-3.5kb-F and BA-3.5kb-R were run on an agarose gel to identify the BCR-ABL sequences in leukemia cells. The results show that the 3.5 kb DNA fragments were only amplified in the CML cells KBM5 and K562. M, DNA marker; 293T, control 293T cells; KBM5 and K562, CML leukemia cell lines. (B)The sequencing results of PCR products. The sequence marked in green is from the *BCR* gene and the red sequence is from the *c-ABL* gene. Highlighted sequences in yellow are *BCR* exons (exons 12, 13, and 14, respectively. The underlined sequences are the BA-3.5kb-F and BA-3.5kb-R primer binding sites. CML, chronic myeloid leukemia; BCR, breakpoint cluster region protein; ABL, Abl Oncogene 1, Receptor Tyrosine Kinase.

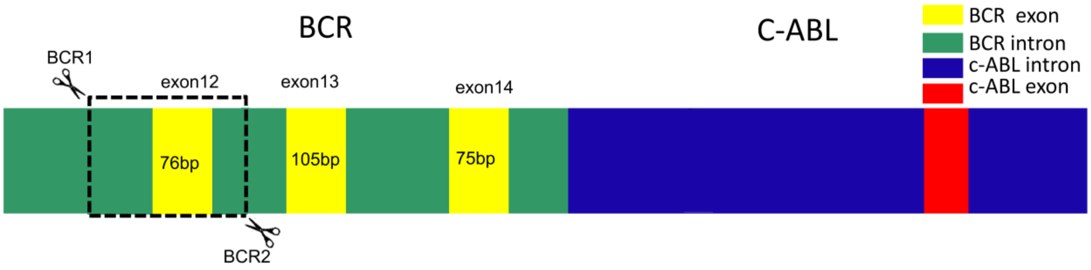

Supplementary Figure S2. Schematic diagram of the disruption of the p210 BCR-ABL genomic sequence using Cas9 combined with two BCR sgRNAs for the targeted deletion of BCR sequences To disrupt the *BCR-ABL* fusion genomic sequence to ablate the coding sequence, we performed targeted deletion of the sequence of *BCR* gene including one exon (exon 12) which contains a 76 bp sequence. This schematic diagram only shows a portion of *BCR-ABL* fusion gene. BCR, breakpoint cluster region protein; ABL, Abl Oncogene 1, Receptor Tyrosine Kinase; Cas9, CRISPR-associated protein 9; sgRNA, single guide RNA.

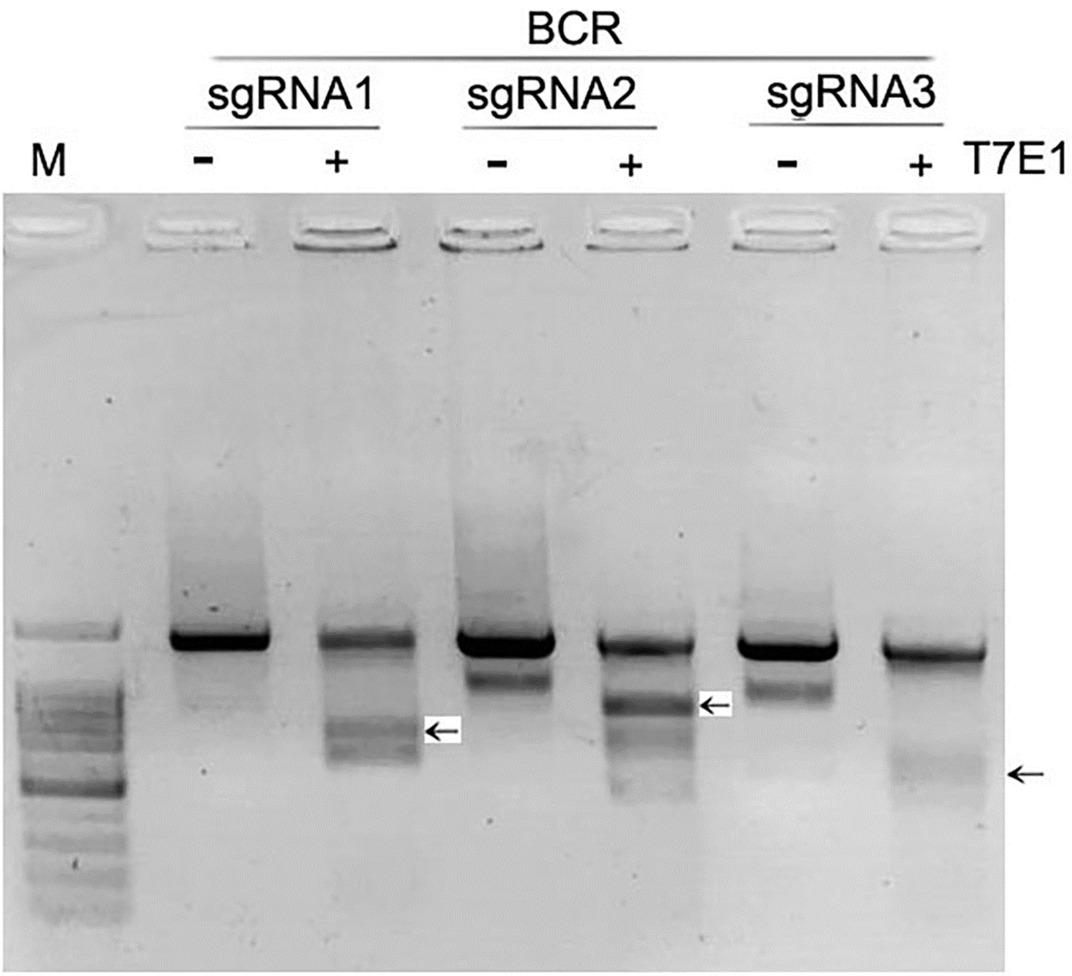

Supplementary Figure S3. The disruption rate in *BCR* loci induced by transfecting the Cas9-GFP/BCR-sgRNA vector into K562 cells The GFP$^+$ cells were sorted by FACS, and the genomic DNA was isolated and amplified using *BCR* primers. The PCR products were used in a disruption efficiency assay by the T7E1 method. "-" represents without T7E1 enzyme; "+" represents plus T7E1 enzyme; Arrowheads indicate cleaved fragments. "M" represents DNA markers. GFP, green fluorescent protein; FACS, fluorescence activated cell sorting; T7E1, T7 Endonuclease I; BCR, breakpoint cluster region protein; Cas9, CRISPR-associated protein 9; sgRNA, single guide RNA.

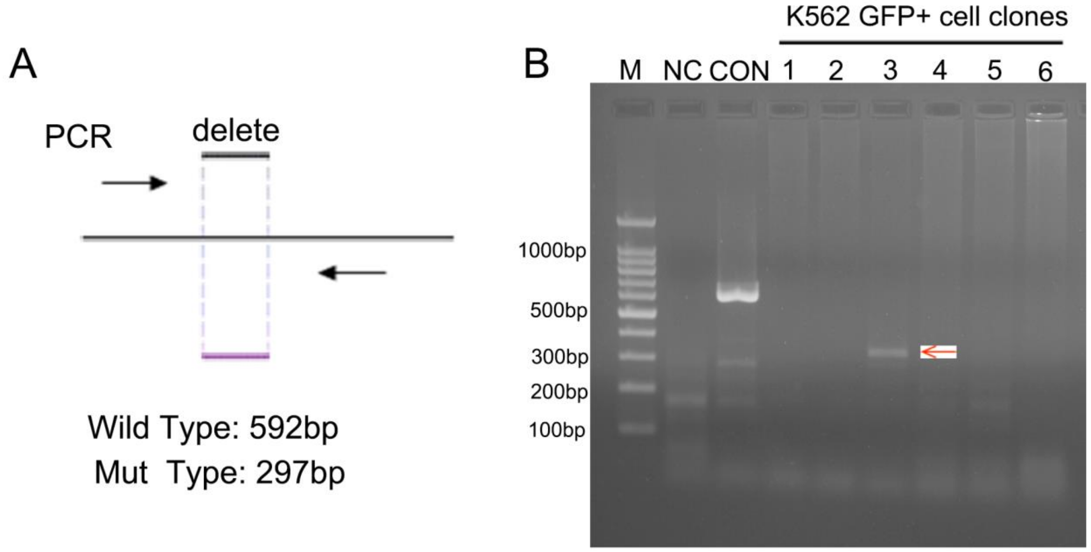

Supplementary Figure S4. Screening of BCR genomic DNA targeted deletion cell clones
After infection by Cas9-P2A-GFP/dual-sgRNA virus (the U6-dual-BCR sgRNA sequence is listed in Table S2), K562 GFP^{+} cells were sorted by FACS. The GFP^{+} cells were cultivated and expanded in 6-well plates. Then, the genomic DNA was isolated, and DNA was amplified using *BCR* primers. The PCR products run through an agarose gel to screen the targeted deletion cell clones. (A) Schematic diagram of using PCR primer pairs to amplify the *BCR* genomic sequence. The targeted deletion clones produce a 297 bp DNA fragment (mutation type); whereas, the wild-type product is 592 bp in length. (B) One of representative positive cell clone producing a 293 bp fragment after PCR. "NC" represents PCR negative control; "CON" represents k562 cells without virus infection; "1, 2, 3, 4, 5, and 6" represents cell clones from GFP+ K562 cells. Arrowhead indicates 297 bp DNA fragment. "M" represents DNA markers. GFP, green fluorescent protein; FACS, fluorescence activated cell sorting; BCR, breakpoint cluster region protein; Cas9, CRISPR-associated protein 9; sgRNA, single guide RNA.

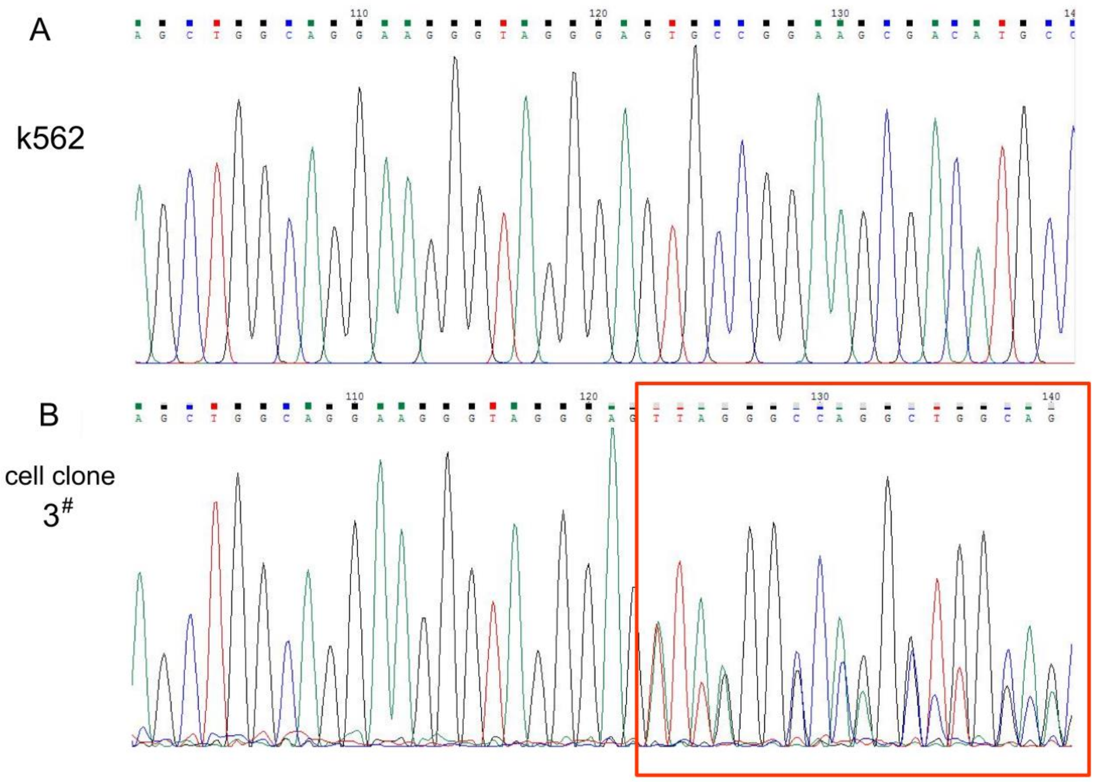

Supplementary Figure S5. DNA sequencing trace of single cell-derived clones Single GFP+ cell-derived clones were used to analyze *BCR* targeted disruption. Genomic DNA from cell clones was PCR amplified, and the PCR products were sequenced directly. Representative DNA sequencing traces of cell clones are shown. Panel A: control K562 cells; Panel B: the cell clones screened out with *BCR* exon 12 deleted. GFP, green fluorescent protein; BCR, breakpoint cluster region protein.

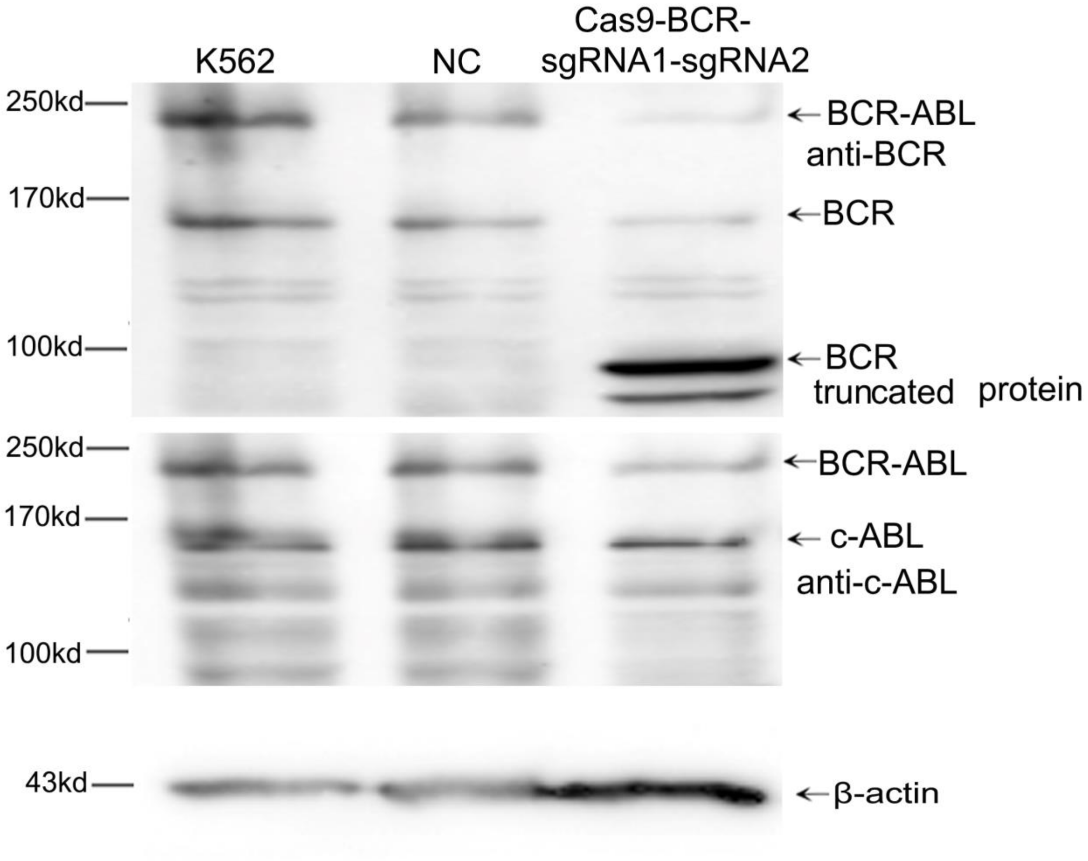

Supplementary Figure S6. Representative western blots for BCR-ABL protein expression in single cell-derived clones Single GFP$^+$ cell-derived clones were used to analyze BCR-ABL fusion protein expression by western blotting. A truncated BCR protein was obviously detected using anti-BCR antibodies. K562; NC (control K562 cells), the k562 cells infected by negative control virus (Cas9-P2A-GFP/control-sgRNA); Cas9-BCR-sgRNA1-sgRNA2, k562 cells infected by virus (Cas9-P2A-GFP/BCR-sgRNA1-BCR-sgRNA2); β-actin was used as the loading control. GFP, green fluorescent protein; BCR, breakpoint cluster region protein; ABL, Abl Oncogene 1, Receptor Tyrosine Kinase; Cas9, CRISPR-associated protein 9; sgRNA, single guide RNA.

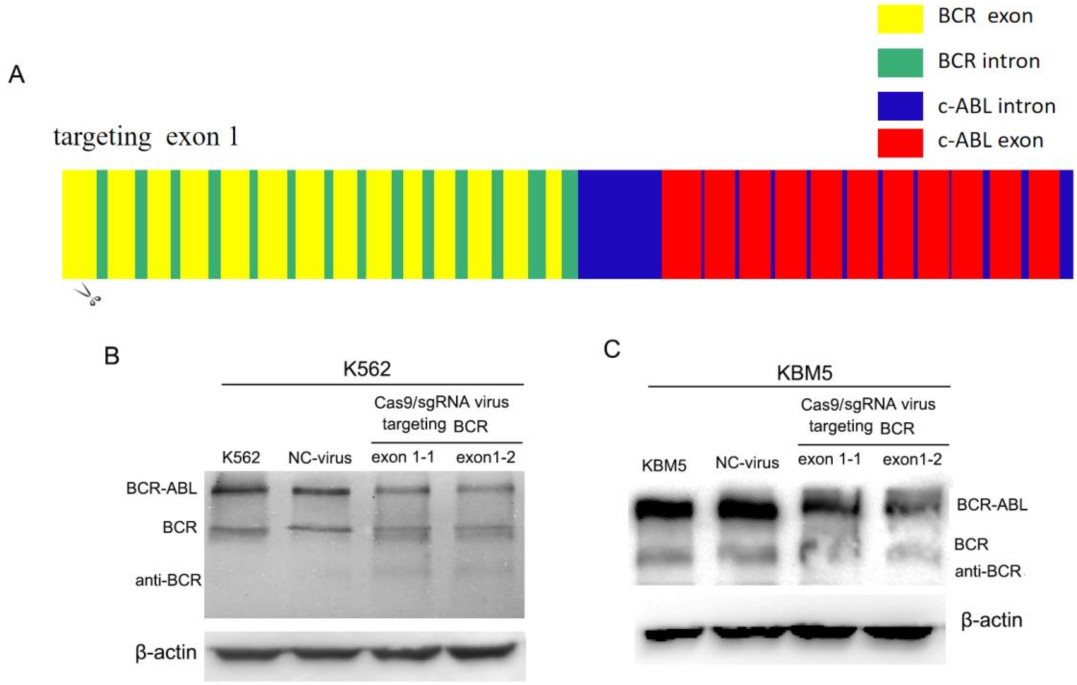

Supplementary Figure S7. The effect of a single sgRNA targeting the first exon of *BCR*
(A) Schematic diagram of targeting the first exon of the *BCR* gene. The effect of a single sgRNA targeting the first exon of *BCR* on BCR-ABL and BCR protein level. After GFP$^+$ cell sorting, the levels of BCR-ABL and BCR proteins were detected by western blotting in K562 cells (B) and KBM5 cells (C). The results showed that the levels of BCR-ABL and BCR proteins had decreased moderately. NC-virus, control lentivirus; exon1-1, virus with sgRNA targeting BCR exon1-1; exon1-2, virus with sgRNA targeting BCR exon1-2; GFP, green fluorescent protein; BCR, breakpoint cluster region protein; ABL, Abl Oncogene 1, Receptor Tyrosine Kinase; Cas9, CRISPR-associated protein 9; sgRNA, single guide RNA.

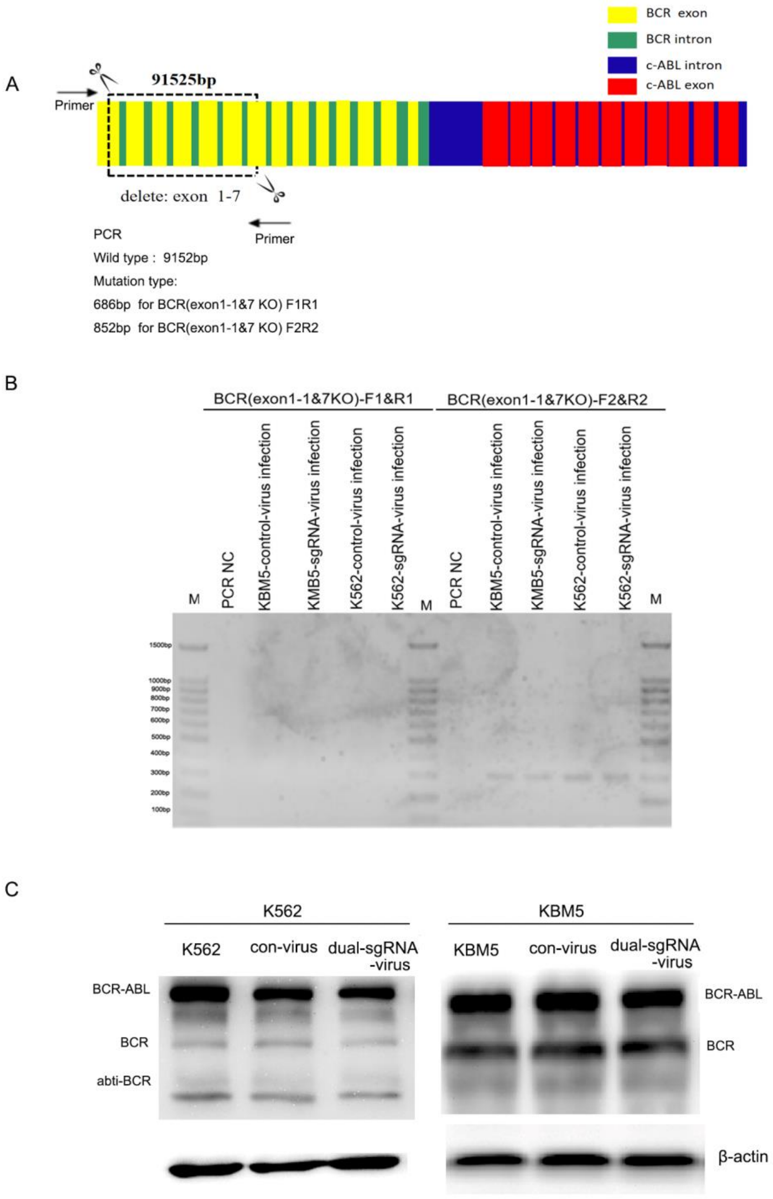

Supplementary Figure S8. The effect of using Cas9/dual-sgRNAs to target the first exon (exon 1) and the seventh exon (exon 7) of *BCR* respectively (A) Schematic diagram of the targeting of exon1 and exon7 of the *BCR* gene using Cas9/dual-sgRNAs. (B) The results of PCR detection by agarose gel electrophoresis after GFP^+ cell sorting. (C) The effect of dual-sgRNAs targeting exon1 and exon 7 of *BCR* on the protein levels of BCR-ABL and BCR. After GFP^+ cell sorting, the levels of BCR-ABL and BCR proteins were detected by western blotting in K562 cells and KBM5 cells. Unfortunately, the target deletion fragment of the genomic DNA between these two sgRNAs was too long; therefore, the results showed that neither the genomic DNA nor the protein expression of BCR-ABL and BCR genes changed significantly in K562 and KBM5 cells. CON-virus, control lentivirus; dual-sgRNA virus, virus with sgRNA targeting BCR exon 1 and exon 7; GFP, green fluorescent protein; BCR, breakpoint cluster region protein; ABL, Abl Oncogene 1, Receptor Tyrosine Kinase; Cas9, CRISPR-associated protein 9; sgRNA, single guide RNA.

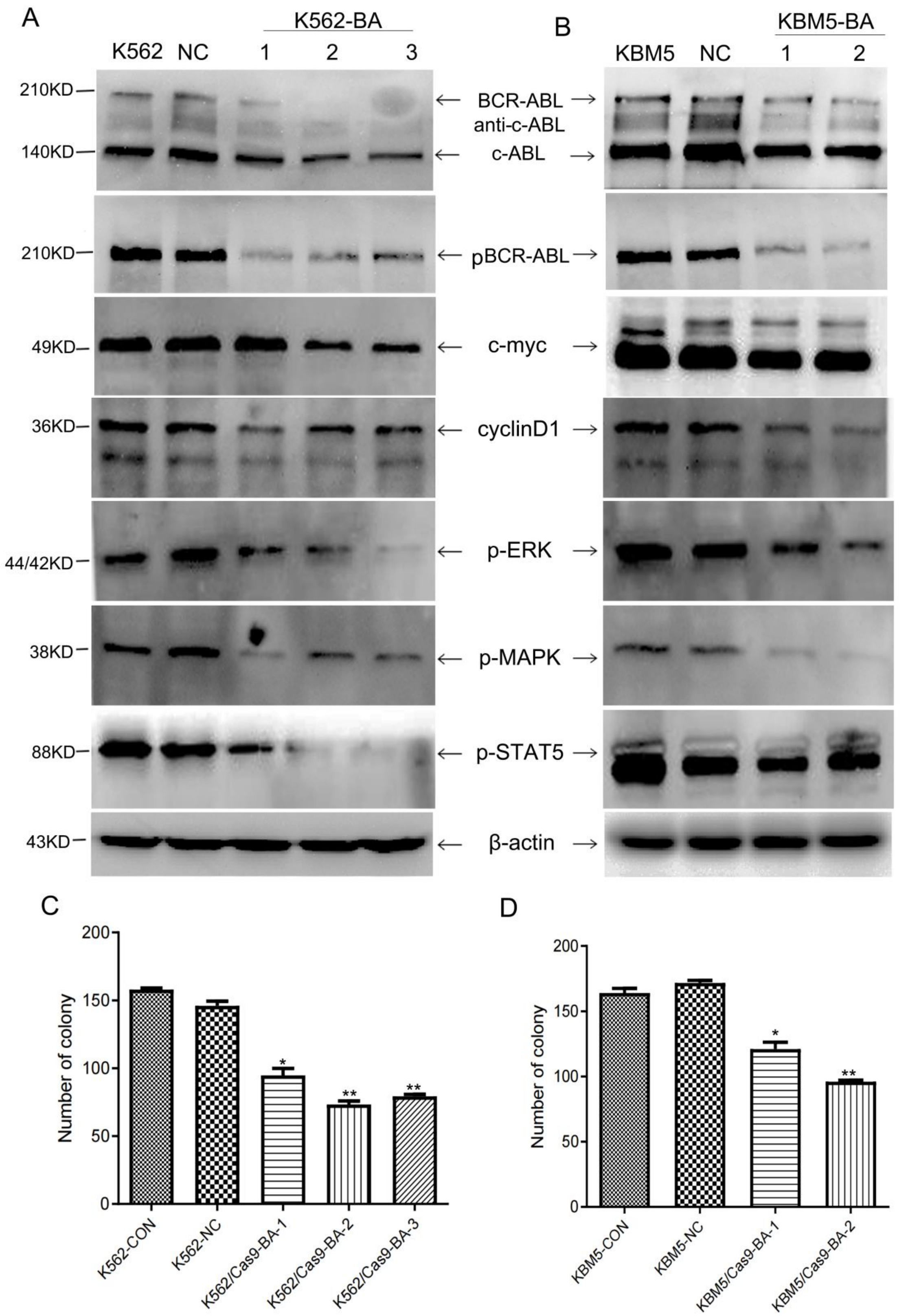

Supplementary Figure S9. The protein levels and cell clone forming abilities of K562-BA and KBM5-BA single cell clones After K562 and KBM5 cells were infected with Cas9-GFP/dual-BA-sgRNAs virus, single GFP^+ cells were sorted and cultivated to form cell clones. The protein expression and cell clone forming abilities of several cell clones was tested. (A,B) The levels of BCR-ABL and related molecules in single GFP^+ cell-derived clones of K562

and KBM5 infected with Cas9-GFP/dual-BA-sgRNA virus. The level of phospho-BCR-ABL and the changes in the levels of its downstream molecules, such as phospho-STAT5, phospho-MAPK, phospho-ERK, c-myc, β-catenin and cyclinD1, were determined in both K562-BA and KBM5-BA cells. (C,D) Cell clone forming abilities of K562-BA and KBM5-BA cells. * $P < 0.05$,** $P < 0.01$. NC, cells infected by control virus (Cas9-P2A-GFP/control-sgRNA); K562-BA& KBM5-BA, single cell-derived cell clones of K562 or KBM5 cells infected with Cas9-dual-sgRNA virus (Cas9-P2A-GFP/sgRNA1(BCR sgRNA1)-sgRNA2 (c-ABLsgRNA1)); GFP, green fluorescent protein; BCR, breakpoint cluster region protein; ABL, Abl Oncogene 1, Receptor Tyrosine Kinase; Cas9, CRISPR-associated protein 9; sgRNA, single guide RNA; p-STAT5, phosphorylated signal transducer and activator of transcription; p-MAPK, phosphorylated mitogen activated protein kinase; p-ERK, phosphorylated extracellular regulated kinase.

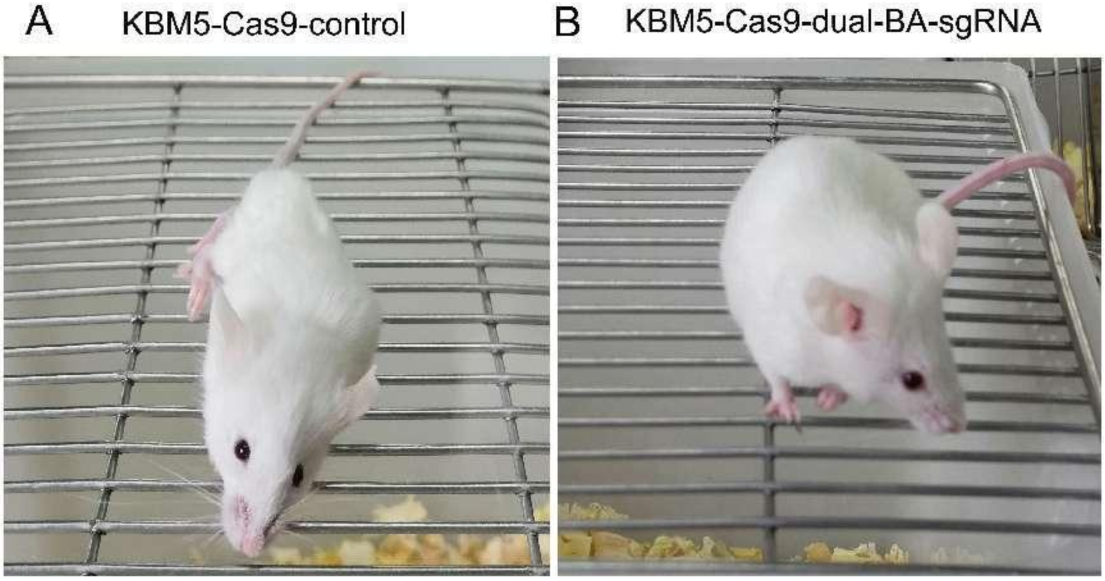

Supplementary Figure S10. NOD-SCID mice after injection with KBM5 GFP$^+$ cells infected with Cas9-P2A-GFP/dual-sgRNAs virus KBM5 GFP$^+$ cells were screened out and injected into NOD-SCID mice by tail vein injection to construct CML mouse model. (A) KBM5-Cas9-contrl, mouse injected with control KBM5 cells infected with control virus (Cas9-P2A-GFP/control-sgRNA); (B) KBM5-Cas9-dual-BA-sgRNA, mouse injected with KBM5 cells infected with Cas9-dual-sgRNA virus (Cas9-P2A-GFP/sgRNA1(BCR sgRNA1)-sgRNA2(c-ABLsgRNA1)). The mouse injected with control KBM5 cells showed the characteristics of CML onset and was dying. However, the mouse injected with KBM5 cells infected with Cas9-dual-sgRNA virus showed no symptoms of leukemia. Nonobese diabetic/severe combined immunodeficiency; GFP, green fluorescent protein; BCR, breakpoint cluster region protein; ABL, Abl Oncogene 1, Receptor Tyrosine Kinase; Cas9, CRISPR-associated protein 9; sgRNA, single guide RNA.

Supplementary Table S1. Primers, oligonucleotides and sequences used in the present study

Name	Sequences (5′⟶3′)
BA-3.5kb-F	TCCTGGTTGCCTAATGGCAGTG
BA-3.5kb-R	TGGTCTCCACTATCAAGGGACA
sgRNA-primer-F (Sac II)	TCCCCGCGGAAGGTCGGGCAGGAAGAGG
sgRNA-primer-R (Nsi I)	CCAATGCATAAAAAAAGCACCGACTCGG
Xho I-IRES-eGFP-F	CCGCTCGAGGCCCCTCTCCCTCCCCCC
Xba I-IRES-eGFP-R	TGCTCTAGATTACTTGTACAGCTCGTCCAT
BCR-F	TCCTGGTTGCCTAATGGCAGTG
BCR-R	GCACAGTGTGAATGCCCAA
BCR-F2	TCCTGGTTGCCTAATGGCAGTG
BCR-R2	GCACAGTGTGAATGCCCAAG
BCRKO-primer-F	GCTAGTGTGGGGCTTGTGAA
BCRKO-primer-R	CTATCGGGATGGCTGTCGC
c-ABL-F	ACTTTCAAAATACCTGCTGCTTCT
c-ABL-R	CGACACTTGAGGAACAAGCC
BAKO-primer-F1	GATCTGGCCCTTGGTACAGG
BAKO-primer-R1	AGACCCCCGGGATTTTACCA
BAKO-primer-F2	GCCTAATGGCAGTGCGTTTT
BAKO-primer-R2	ACCAGACCCCCGGGATTTTA
BCR(exon1-1&7KO)-F1	CAACGACAAAGAGGTGTCGGA
BCR(exon1-1&7KO)-F2	CGACAAAGAGGTGTCGGACC
BCR(exon1-1&7KO)-R1	TCGGGGTGTGATCTCCTCAT
BCR(exon1-1&7KO)-R2	GGCCAATCTCACCAATCCCA
BCR Cas9 target site 1	GCTGGCAGGAAGGGTAGGGAGTGC**CGG**AAGCGAC
BCR Cas9 target site 2	GAGGGGCTGGGCTTCAAACCATTA**GGG**CCAGGCTG
BCR Cas9 target site 3	TTGCGTAGCCAGGGCGGAGATAAC**TGG**GTGTGTTC
BCR Cas9 target site 4 (exon1-1)	GATGGAGCGCAAAAAGTCCCAGCA**CGG**CGCGGGC
BCR Cas9 target site5 (exon1-2)	GCCCTACCAGAGCATCTACGTCGG**GGG**CATGATG
BCR Cas9 target site 6 (exon7)	TACAAGCCTGTGGACCGTGTGACG**AGG**AGCACGC
c-ABL Cas9 target site 1	CACTCAGTACTTGTCCCTTGATAG**TGG**AGACCATG
c-ABL Cas9 target site 2	CAGTGTGGAATGGGTCCAGGGAAC**CGG**CTGAGGCT

hU6-sgRNA1 (BCR sgRNA1)-mU6-sgRNA2 (BCR sgRNA2)	**ggtacc**GAGGGCCTATTTCCCATGATTCCTTCATATTTGCATATACG<br>ATACAAGGCTGTTAGAGAGATAATTAGAATTAATTTGACTGTAAAC<br>ACAAAGATATTAGTACAAAATACGTGACGTAGAAAGTAATAATTTC<br>TTGGGTAGTTTGCAGTTTTAAAATTATGTTTTAAAATGGACTATCAT<br>ATGCTTACCGTAACTTGAAAGTATTTCGATTTCTTGGCTTTATATAT<br>CTTGTGGAAAGGACGAAACACCGGCAGGAAGGGTAGGGAGTG<br>CGTTTCAGAGCTATGCTGGAAACAGCATAGCAAGTTGAAATAAGG<br>CTAGTCCGTTATCAACTTGAAAAAGTGGCACCGAGTCGGTGCTT<br>TTTTCTCGAGGATCCGACGCCGCCATCTCTAGGCCCGCGCCGG<br>CCCCCTCGCACAGACTTGTGGGAGAAGCTCGGCTACTCCCCTG<br>CCCCGGTTAATTTGCATATAATATTTCCTAGTAACTATAGAGGCTTA<br>ATGTGCGATAAAAGACAGATAATCTGTTCTTTTTAATACTAGCTACA<br>TTTTACATGATAGGCTTGGATTTCTATAAGAGATACAAATACTAAAT<br>TATTATTTTAAAAAACAGCACAAAAGGAAACTCACCCTAACTGTAA<br>AGTAATTGTGTGTTTTGAGACTATAAATATCCCTTGGAGAAAAGCC<br>TTGTTTGGGCTGGGCTTCAAACCATTAGTTTCAGAGCTATGCTG<br>GAAACAGCATAGCAAGTTGAAATAAGGCTAGTCCGTTATCAACTT<br>GAAAAAGTGGCACCGAGTCGGTGCTTTTTT**gaattc**
hU6-sgRNA1 (BCR sgRNA1) - mU6-sgRNA2 (c-ABL sgRNA1)	**ggtacc**GAGGGCCTATTTCCCATGATTCCTTCATATTTGCATATACG<br>ATACAAGGCTGTTAGAGAGATAATTAGAATTAATTTGACTGTAAAC<br>ACAAAGATATTAGTACAAAATACGTGACGTAGAAAGTAATAATTTC<br>TTGGGTAGTTTGCAGTTTTAAAATTATGTTTTAAAATGGACTATCAT<br>ATGCTTACCGTAACTTGAAAGTATTTCGATTTCTTGGCTTTATATAT<br>CTTGTGGAAAGGACGAAACACCGGCAGGAAGGGTAGGGAGTG<br>CGTTTCAGAGCTATGCTGGAAACAGCATAGCAAGTTGAAATAAGG<br>CTAGTCCGTTATCAACTTGAAAAAGTGGCACCGAGTCGGTGCTT<br>TTTTCTCGAGGATCCGACGCCGCCATCTCTAGGCCCGCGCCGG<br>CCCCCTCGCACAGACTTGTGGGAGAAGCTCGGCTACTCCCCTG<br>CCCCGGTTAATTTGCATATAATATTTCCTAGTAACTATAGAGGCTTA<br>ATGTGCGATAAAAGACAGATAATCTGTTCTTTTTAATACTAGCTACA<br>TTTTACATGATAGGCTTGGATTTCTATAAGAGATACAAATACTAAAT<br>TATTATTTTAAAAAACAGCACAAAAGGAAACTCACCCTAACTGTAA<br>AGTAATTGTGTGTTTTGAGACTATAAATATCCCTTGGAGAAAAGCC<br>TTGTTTGCAGTACTTGTCCCTTGATAGGTTTCAGAGCTATGCTGG<br>AAACAGCATAGCAAGTTGAAATAAGGCTAGTCCGTTATCAACTTG<br>AAAAAGTGGCACCGAGTCGGTGCTTTTTT**gaattc**
hU6-sgRNA (BCRexon1-1)	ggtaccGAGGGCCTATTTCCCATGATTCCTTCATATTTGCATATACGA<br>TACAAGGCTGTTAGAGAGATAATTAGAATTAATTTGACTGTAAACA<br>CAAAGATATTAGTACAAAATACGTGACGTAGAAAGTAATAATTTCTT<br>GGGTAGTTTGCAGTTTTAAAATTATGTTTTAAAATGGACTATCATAT<br>GCTTACCGTAACTTGAAAGTATTTCGATTTCTTGGCTTTATATATCT<br>TGTGGAAAGGACGAAACACCGGAGCGCAAAAAGTCCCAGCAGT<br>TTCAGAGCTATGCTGGAAACAGCATAGCAAGTTGAAATAAGGCTA<br>GTCCGTTATCAACTTGAAAAAGTGGCACCGAGTCGGTGCTTTTTT

	gaattc
hU6-sgRNA (BCR exon1-2)	ggtaccGAGGGCCTATTTCCCATGATTCCTTCATATTTGCATATACGATACAAGGCTGTTAGAGAGATAATTAGAATTAATTTGACTGTAAACACAAAGATATTAGTACAAAATACGTGACGTAGAAAGTAATAATTTCTTGGGTAGTTTGCAGTTTTAAAATTATGTTTTAAAATGGACTATCATATGCTTACCGTAACTTGAAAGTATTTCGATTTCTTGGCTTTATATATCTTGTGGAAAGGACGAAACACCGTACCAGAGCATCTACGTCGGGTTTCAGAGCTATGCTGGAAACAGCATAGCAAGTTGAAATAAGGCTAGTCCGTTATCAACTTGAAAAAGTGGCACCGAGTCGGTGCTTTTTTgaattc
hU6-sgRNA1 (BCRexon1-1)-mU6-sgRNA2 (BCRexon7)	ggtaccGAGGGCCTATTTCCCATGATTCCTTCATATTTGCATATACGATACAAGGCTGTTAGAGAGATAATTAGAATTAATTTGACTGTAAACACAAAGATATTAGTACAAAATACGTGACGTAGAAAGTAATAATTTCTTGGGTAGTTTGCAGTTTTAAAATTATGTTTTAAAATGGACTATCATATGCTTACCGTAACTTGAAAGTATTTCGATTTCTTGGCTTTATATATCTTGTGGAAAGGACGAAACACCGGAGCGCAAAAAGTCCCAGCAGTTTCAGAGCTATGCTGGAAACAGCATAGCAAGTTGAAATAAGGCTAGTCCGTTATCAACTTGAAAAAGTGGCACCGAGTCGGTGCTTTTTTCTCGAGGATCCGACGCCGCCATCTCTAGGCCCGCGCCGGCCCCCTCGCACAGACTTGTGGGAGAAGCTCGGCTACTCCCCTGCCCCGGTTAATTTGCATATAATATTTCCTAGTAACTATAGAGGCTTAATGTGCGATAAAAGACAGATAATCTGTTCTTTTTAATACTAGCTACATTTTACATGATAGGCTTGGATTTCTATAAGAGATACAAATACTAAATTATTATTTTAAAAAACAGCACAAAAGGAAACTCACCCTAACTGTAAAGTAATTGTGTGTTTTGAGACTATAAATATCCCTTGGAGAAAAGCCTTGTTTGAGCCTGTGGACCGTGTGACGGTTTCAGAGCTATGCTGGAAACAGCATAGCAAGTTGAAATAAGGCTAGTCCGTTATCAACTTGAAAAAGTGGCACCGAGTCGGTGCTTTTTTgaattc

Supplementary Table S2. Clinical features of patients with CML

No.	Age	sex	WBC (10^9/L)	Hb (g/L)	Plt (10^9/L)	Splenomegaly	Protein	Disease	Therapy
1	55	F	189	105	448	+	P210	CML-CP	Imatinib
2	65	F	118.9	57	31	+	P210	CML-BP(ALL)	Imatinib
3	53	F	75.65	114	872	+	P210	CML-CP	Imatinib
4	44	M	301.6	67	485	+	P210	CML-CP	Imatinib
5	53	F	227	79	491	+	P210	CML-CP	Imatinib
6	54	F	95.59	109	363	-	P210	CML-CP	Imatinib

WBC: white blood cells; Hb: hemoglobin; Plt: Platelets; F: female; M: male; CML-CP: chronic phase; CML-BP: blastic phase; BP(ALL): B-cell precursor acute lymphoblastic leukemia.

Supplementary Table S3. Candidate off-target InDels in the K562 dual BA sgRNA sample identified by GATK Variant Calling

Chr.	Position	Change	Mut.	Mismatches	Gene Symbol
chr1	192830941	A>AGGAG	I	3	
chr1	9932345	C>CA	I	5	LZIC
chr1	85167213	C>CA	I	5	SYDE2
chr1	10386266	C>CAA	I	5	
chr1	227383268	C>CT	I	5	
chr1	37427328	CA>C	D	5	
chr1	206776909	CAA>C	D	5	IL19
chr1	159205214	CT>C	D	5	ACKR1
chr1	66307698	G>GC	I	5	PDE4B
chr1	66307703	T>TGCCTCCAGTAGCCAGGAAGGAAATG	I	5	PDE4B
chr1	66307700	T>TTGCC	I	5	PDE4B
chr1	117429842	TGGCCGGGCGGGG>T	D	5	MAN1A2
chr10	114514298	C>CA	I	5	ABLIM1
chr10	7287077	C>CTT	I	5	SFMBT2
chr11	11620330	C>CGT	I	5	GALNT18
chr11	61845784	CA>C	D	5	FADS2
chr11	63790555	G>GAA	I	4	
chr11	4098458	T>TA	I	4	RRM1
chr12	21089723	AT>A	D	4	SLCO1B3SLCO1B7
chr12	30796783	C>CA	I	5	LINC00941
chr12	103013305	C>CA	I	5	
chr12	95828074	C>CGGAA	I	5	SNRPFDT
chr12	713344	CA>C	D	5	LOC101929432
chr12	105199990	G>GT	I	5	LOC105369953
chr12	30796778	GC>G	D	5	LINC00941
chr13	52486565	CA>C	D	5	LINC00345
chr14	96616863	AG>A	D	5	
chr14	64602011	CT>C	D	5	
chr14	91493773	CT>C	D	5	PPP4R3A

chr15	65049336	AAG>A	D	5	RASL12
chr15	64016803	AGGGAAG>A	D	5	DAPK2
chr15	90673541	C>CCCTTCCTT	I	5	CRTC3AS1
chr15	63530326	C>CT	I	5	USP3
chr15	85252216	G>GT	I	5	
chr15	83069288	T>TG	I	5	
chr16	7304214	C>CAG	I	5	RBFOX1
chr16	997700	G>GCCGTCA	I	5	LOC107984898
chr16	50152772	G>GT	I	4	
chr16	84028570	GA>G	D	5	SLC38A8
chr17	9542004	ATT>A	D	4	STX8
chr17	32443808	CT>C	D	4	
chr17	45742047	G>GGA	I	5	LINC02210CRHR1
chr17	81004216	GGGCGGCGGCGCGGGAAGCA>G	D	5	LOC100129503
chr18	58853265	A>AAG	I	5	
chr18	4214311	AT>A	D	5	DLGAP1
chr19	7149936	A>AGAAGGAAGGAAGGAAGGAAGGAAGGAAG	I	5	INSR
chr19	17970886	AT>A	D	5	KCNN1
chr19	52820662	C>CCTCA	I	5	ZNF28
chr19	10842736	CA>C	D	5	C19orf38
chr19	12485397	CT>C	D	5	
chr19	7149908	G>GGAAA	I	5	INSR
chr2	197410501	A>AT	I	5	SF3B1
chr2	23877067	C>CA	I	5	ATAD2B
chr2	182577931	C>CGGAAGGAA	I	5	
chr2	32065973	C>CTT	I	5	SPAST
chr2	238980499	C>CTT	I	5	
chr2	84951147	CTT>C	D	5	LOC105374838
chr2	238234564	G>GC	I	4	
chr2	205734170	TTA>T	D	5	NRP2
chr20	3789988	C>CA	I	5	CDC25B
chr20	49071967	CA>C	D	4	CSE1L
chr20	37856200	CAAA>CAA	D	5	CTNNBL1
chr20	20847794	G>GT	I	5	
chr20	35090180	TAA>TA	D	5	TRPC4AP
chr21	23891100	A>AAT	I	5	

chr21	10430799	A>ACTCTCATCTTTTTCCC	I	5	BAGE2
chr21	45990474	A>AGATGGGGAGGGACGGAGTGGACGGCGTGAAGGTGACC	I	5	COL6A1
chr21	20640861	C>CA	I	5	
chr21	34053863	CA>CAA	I	5	
chr21	45990464	G>GAATGGGGC	I	5	COL6A1
chr21	8213184	G>GT	I	4	RNA45SN2
chr22	48667087	A>AGGTAG	I	5	TAFA5
chr22	35088874	A>ATC	I	5	
chr22	44289466	A>ATG	I	5	SHISAL1
chr22	32315811	AAAAAG>A	D	5	SLC5A4
chr22	25363526	C>CA	I	5	
chr22	49648856	G>GC	I	5	MIR3667HG
chr3	108844319	AT>A	D	5	TRAT1
chr3	29124484	C>CTT	I	5	
chr3	141821512	CAA>CA	D	5	
chr3	125765278	CTCCCTGGCCTCCTCTGCT>C	D	5	
chr3	125765249	G>GCC	I	5	
chr3	125765252	TAG>T	D	5	
chr4	3973435	A>AGG	I	5	
chr4	157220652	A>ATG	I	5	GRIA2
chr4	1247767	AG>A	D	5	CTBP1
chr4	8553771	CT>C	D	5	
chr4	152938190	CT>C	D	4	FHDC1
chr4	150848562	G>GA	I	5	LRBA
chr4	182917480	G>GGGGA	I	5	DCTD
chr4	9135163	GTCTC>G	D	5	LOC105369250
chr5	70074643	CA>C	D	5	SMN2
chr5	53759804	CT>C	D	5	
chr6	168044205	A>AGAGGGTGAGGGGTGCTAGTGACCGGCTGCTGACCCTCCCG	I	5	KIF25
chr6	43546790	G>GA	I	5	POLR1C
chr6	122850303	GGGTTTTCTTTTT>G	D	5	

chr6	7108316	T>TTCCTCCTCC	I	5	RREB1
chr6	14624415	TC>T	D	5	LOC101928354
chr6	33566452	TTCCCTCCC>T	D	4	
chr7	99888342	A>ACCCC	I	5	
chr7	5998989	CA>C	D	5	PMS2
chr7	129566902	CCA>C	D	5	
chr7	157357212	G>GTCCT	I	5	DNAJB6
chr7	99888349	T>TACCTCCCTCCCGGACGGGGCGGCTGGCTGGGCGGGGGGCTGACCC	I	5	
chr7	157357233	TCC>T	D	5	DNAJB6
chr8	119865168	C>CT	I	5	
chr9	133565839	C>CCA	I	5	ADAMTSL2
chr9	93959448	C>CT	I	5	
chr9	81572930	T>TA	I	5	
chrX	115725239	AAG>A	D	5	
chrX	119488887	AT>A	D	5	
chrX	130127036	GGT>G	D	5	RAB33A
chrX	13142854	GT>G	D	3	

Chr. = Chromosome. Mut. = mutation: I = insertion, D = deletion. Change = sequence change in K562-BA sgRNA1 (BCR sgRNA1) relative to K562.

Supplementary Table S4. Candidate off-target InDels in the K562 dual BA sgRNA sample identified by GATK Variant Calling

Chr.	Position	Change	Mut.	Mismatches	Gene Symbol
chr1	40551589	A>AT	I	4	
chr1	238387314	AG>A	D	5	LOC105373220
chr1	212069548	AT>A	D	4	DTL
chr1	152228502	C>CA	I	5	
chr1	185550298	C>CA	I	5	LOC107985239
chr1	190706351	C>CA	I	5	LINC01720
chr1	78251038	C>CGA	I	5	MGC27382
chr1	150727937	C>CTTTT	I	5	
chr1	212632570	CA>C	D	4	LINC02773
chr1	64994963	CAA>C	D	5	JAK1
chr1	179347357	CAA>C	D	5	SOAT1
chr1	8093459	CAA>CA	D	5	
chr1	9059536	CT>C	D	5	SLC2A5
chr1	21014635	CT>C	D	5	EIF4G3
chr1	32144656	CT>C	D	5	KPNA6
chr1	150291493	CT>C	D	5	
chr1	242757815	G>GAA	I	5	
chr1	223479588	T>TCC	I	5	
chr1	62212779	TAAAAA>TAAAA	D	5	
chr1	70500248	TTGTGTG>TTGTG	D	5	
chr10	4863726	AAATCAATGAATCCAGGAGCT> AAATCAATGAATCCAGGAGCTG	I	5	AKR1E2
chr10	61848631	AT>A	D	5	LINC02625
chr10	101329191	AT>A	D	4	
chr10	89739660	C>CT	I	5	KIF20B
chr10	94309317	C>CT	I	5	PLCE1
chr10	3653431	C>CTTTCTTTTCTTTTCT	I	4	LOC105376360
chr10	129946014	CA>C	D	5	EBF3
chr10	69794158	CGT>C	D	5	
chr10	101624091	GAC>G	D	5	FBXW4
chr10	32579236	GT>G	D	4	CCDC7
chr10	93114034	GT>G	D	5	
chr10	113163009	T>TA	I	5	TCF7L2
chr10	107301942	T>TAC	I	5	
chr10	44077642	T>TC	I	5	
chr10	34099951	TAC>TACAC	I	5	
chr10	44077621	TG>T	D	5	
chr11	121341611	AAAG>A	D	5	
chr11	40160178	AT>A	D	5	LRRC4C

chr11	117889338	AT>A	D	5	
chr11	84061851	C>CA	I	4	DLG2
chr11	118301528	C>CA	I	5	
chr11	129401955	C>CA	I	5	BARX2
chr11	32233583	C>CAA	I	5	
chr11	112695988	C>CT	I	5	
chr11	10532526	CA>C	D	5	RNF141
chr11	28758945	CA>C	D	5	
chr11	37551184	CA>C	D	5	
chr11	108963818	CA>CAA	I	5	
chr11	2709220	G>GC	I	4	KCNQ1
chr11	109218686	G>GT	I	5	
chr11	8281128	GAA>GA	D	4	
chr11	44273190	TA>T	D	5	ALX4
chr12	120573585	C>CA	I	5	RNF10
chr12	8643181	C>CTT	I	5	
chr12	52852194	CT>C	D	5	
chr12	2951126	CTT>C	D	5	
chr12	30737598	CTTT>CTT	D	5	CAPRIN2
chr12	18803168	GA>G	D	5	LOC102724227
chr12	94005280	GT>G	D	5	LOC105369912
chr12	105588407	GT>G	D	5	
chr12	131239346	TA>T	D	5	LOC105370082
chr13	56475813	A>AT	I	4	LOC105370214
chr13	40848282	AAC>A	D	5	
chr13	88456957	C>CA	I	5	
chr13	89668167	C>CAA	I	5	
chr13	46355294	CT>C	D	5	RUBCNL
chr13	62252459	CT>C	D	5	
chr13	94024102	G>GAC	I	5	GPC6
chr14	49787271	AAATACC>A	D	5	NEMF
chr14	24745913	CA>C	D	5	
chr14	32608499	CA>C	D	5	AKAP6
chr14	56749218	CT>C	D	5	
chr14	65591840	CT>C	D	5	FUT8
chr14	70614115	CT>C	D	5	TTC9DT
chr14	68595951	CTT>C	D	5	RAD51B
chr14	89791128	CTT>C	D	5	
chr14	61263847	TTG>T	D	5	PRKCH
chr15	28517042	A>AT	I	5	
chr15	97459405	CGTGT>C	D	5	LINC02254
chr15	23390057	CT>C	D	5	
chr15	34863098	CT>C	D	5	AQR

chr15	89081001	CT>C	D	4	CARMAL
chr15	93231304	G>GGA	I	5	LOC105370982
chr15	25490522	GGCTGGGCGCGGTGGCTCACGCCTGTAATCCCAGCACTTTGGGAGGCCGAGGTGGGCGGATCACGAGGTC>G	D	5	
chr15	73940453	GT>G	D	4	LOXL1
chr15	31902893	T>TTTG	I	5	
chr16	74819204	A>AT	I	5	
chr16	81232726	CA>C	D	5	
chr16	333435	GA>G	D	5	AXIN1
chr16	69802604	GT>G	D	4	WWP2
chr16	22344979	TA>T	D	4	
chr16	71137932	TAC>T	D	5	HYDIN
chr17	39476064	A>AT	I	4	CDK12
chr17	19558470	AT>A	D	5	SLC47A1
chr17	7157510	C>CA	I	5	
chr17	17137512	C>CA	I	4	MPRIP
chr17	74673407	C>CA	I	5	RAB37
chr17	11984201	G>GA	I	5	ZNF18
chr17	16199204	GAAAAA>GAAAA	D	5	NCOR1
chr17	10888524	GGT>G	D	5	
chr17	81009727	TAA>TA	D	5	
chr17	13805000	TTC>T	D	5	LOC100506974
chr18	50842568	ATTTT>ATT	D	5	
chr18	30425226	C>CA	I	4	
chr18	35953730	C>CA	I	5	LOC105372066
chr18	49767053	CGTGTGTGTGTGTGTGTGTGTGTGTGTGTGT>CGTGTGTGTGTGTGTGTGTGTGTGTGTGTGTGT	I	5	
chr18	27740663	CT>C	D	5	
chr19	37034062	A>AT	I	5	ZNF420
chr19	15614434	AG>A	D	5	
chr19	7398690	C>CA	I	5	ARHGEF18
chr19	43548766	C>CA	I	5	XRCC1
chr19	51618792	C>CAA	I	5	SIGLEC5
chr19	6208065	CAA>CA	D	5	
chr19	37812514	CT>C	D	5	LOC105372394
chr19	13801155	G>GA	I	5	ZSWIM4
chr19	15614428	G>GAAA	I	5	
chr19	16574921	GT>G	D	5	MED26
chr19	49237208	GTATATATA>GTATATATATA	I	5	LOC107985340

chr2	170902858	A>AT	I	4	
chr2	11273094	C>CA	I	5	ROCK2
chr2	44046899	C>CT	I	5	
chr2	66605436	C>CTTT	I	5	LINC01798
chr2	24829022	C>CTTTCT	I	5	ADCY3
chr2	206996852	CA>C	D	5	
chr2	14410103	CA>CAA	I	5	
chr2	184736858	CT>C	D	5	ZNF804A
chr2	207539044	CT>C	D	5	CREB1
chr2	225499319	CT>C	D	5	NYAP2
chr2	49795072	CT>CTT	I	5	
chr2	7557723	G>GGT	I	5	
chr2	98893202	G>GGTCACTAGATCCA	I	5	CRACDL
chr2	29256609	G>GT	I	5	ALK
chr2	69866249	GA>G	D	5	GMCL1
chr2	155239659	GAA>GA	D	5	
chr2	162492865	GTGTT>G	D	5	KCNH7
chr2	13035215	T>TTA	I	5	LOC105373436
chr2	52413380	TA>T	D	5	
chr2	98224753	TAA>TA	D	5	VWA3B
chr2	154736374	TAAAAAAAAAAAAAAAAAAAAA AA>T	D	5	KCNJ3
chr2	160800584	TAAAG>T	D	5	
chr2	24829018	TTTTC>T	D	5	ADCY3
chr20	42870954	C>CT	I	5	PTPRT
chr20	18513342	CA>C	D	5	SEC23B
chr20	43672427	CA>C	D	5	MYBL2
chr20	39382009	CT>C	D	5	
chr20	42272721	G>GCA	I	4	PTPRT
chr20	15288867	G>GTCTA	I	5	MACROD2
chr20	57491479	GAA>G	D	5	
chr20	22381563	T>TTC	I	5	
chr20	43551366	TA>T	D	5	
chr21	28112500	C>CAA	I	5	LINC01697
chr21	43359654	CCA>C	D	5	LINC01679
chr21	31269671	G>GT	I	5	TIAM1
chr21	33752824	TAA>T	D	5	ITSN1
chr22	29368756	C>CA	I	5	AP1B1
chr22	40210017	C>CA	I	5	TNRC6B
chr22	27924294	G>GT	I	5	TTC28AS1
chr22	27151572	GTT>G	D	4	
chr22	43920636	TA>T	D	4	
chr22	32869330	TTCTCTCTC>TTCTCTCTCTC	I	5	SYN3

chr3	47212727	C>CA	I	5	KIF9AS1
chr3	155503537	C>CT	I	5	PLCH1
chr3	25971205	C>CTG	I	4	
chr3	102362619	CA>C	D	5	
chr3	196322973	CA>C	D	5	TM4SF19DYNLT2B
chr3	196648766	CA>C	D	5	NRROS
chr3	183871517	CAAA>C	D	5	PARL
chr3	45695997	CTT>C	D	4	SACM1L
chr3	66624493	CTT>CTTT	I	4	
chr3	180322854	G>GGA	I	5	
chr3	59857710	T>TTTTTG	I	4	FHIT
chr3	63333336	TA>T	D	5	SYNPR
chr3	106391104	TA>T	D	5	LOC101929485
chr3	131628837	TCA>T	D	5	CPNE4
chr4	93047882	AC>A	D	5	GRID2
chr4	38488439	C>CT	I	5	LINC01258
chr4	40993361	C>CT	I	5	APBB2
chr4	26076401	CA>C	D	5	LINC02357
chr4	156793534	CATAT>CAT	D	5	PDGFC
chr4	113008109	CT>C	D	5	ANK2
chr4	37188602	GA>G	D	5	
chr4	49591813	GT>G	D	5	
chr4	79520372	GT>G	D	5	LINC00989
chr4	112274150	TA>T	D	4	
chr4	104406026	TA>TAA	I	5	
chr5	16635575	ATTTT>A	D	5	
chr5	50378656	C>CA	I	5	
chr5	106369274	C>CA	I	5	
chr5	65849875	C>CT	I	5	
chr5	80053406	C>CTT	I	5	THBS4
chr5	76623936	C>CTTTTTT	I	5	IQGAP2
chr5	32298952	CA>C	D	4	MTMR12
chr5	42389639	CA>CAA	I	5	
chr5	59756810	CT>C	D	4	PDE4D
chr5	98251527	G>GA	I	5	
chr5	150955089	G>GT	I	5	
chr5	105555647	GT>G	D	5	
chr5	94104343	T>TA	I	5	FAM172A
chr5	126059141	T>TA	I	5	
chr5	179676225	T>TAC	I	5	
chr5	30125200	T>TTC	I	5	
chr6	25343316	C>CT	I	4	CARMIL1
chr6	26934819	C>CT	I	5	

chr6	87092742	C>CT	I	5	CGA
chr6	111409360	C>CT	I	5	REV3L
chr6	105008643	CA>C	D	4	LIN28B
chr6	159625213	CAAAAA>CAAAAAA	I	5	
chr6	161841353	CT>C	D	5	PRKN
chr6	106682305	T>TA	I	5	
chr6	106682310	TATTTA>T	D	5	
chr7	127774580	A>AT	I	5	SND1
chr7	54221276	C>CA	I	5	
chr7	149231446	C>CA	I	5	
chr7	7359290	CA>C	D	5	COL28A1
chr7	98328679	CA>C	D	5	BAIAP2L1
chr7	130399819	CAAA>CA	D	5	CEP41
chr7	28557020	CCTAGGGATACTAACAGCTCCCTGCTGAAACTGGCCCCAGGGCTTTCCTACAATCTGTTCCCACCTTTGCAAAAAGTCTCTTTATTAAATGCTCCTCACATTACCCAGTTTGAAGGTA>C	D	5	CREB5
chr7	97086664	CT>C	D	4	
chr7	122447031	CT>C	D	5	CADPS2
chr7	62268709	G>GA	I	5	
chr7	57045464	G>GTA	I	5	
chr7	483641	GGGTGGATC>G	D	5	
chr7	483643	GTGGATAGTTAGATGAATGACAGATGGTTGGACAGATGGGTGGATGGGTAGGTGGGTGGATA>G	D	5	
chr7	136576161	TGGGAGGGCCTTGCTCCAAAATCCTAGAGGCCCACTAGGCGTTATGCCCCTTTTTTG>TGGGAGGGCCTTGCTCCAAAATCCAAATAGGCCCACTAGGCGTTATGCCCCTTTTTTG	I	5	
chr7	57045444	TTGTGTGTGTG>TTGTGTGTG	D	5	
chr8	73063076	A>AT	I	5	
chr8	97317010	A>AT	I	5	LOC101927066
chr8	69257412	A>ATT	I	5	
chr8	73154737	CA>C	D	5	
chr8	17100209	CT>C	D	5	MICU3
chr8	42241224	CTT>C	D	5	
chr8	118618028	G>GT	I	5	SAMD12
chr8	586747	GT>G	D	5	
chr8	43505138	GT>G	D	5	LOC105379397

chr8	10313467	TA>T	D	5	MSRA
chr8	26748750	TA>T	D	5	ADRA1A
chr8	72628442	TGTGA>T	D	5	KCNB2
chr9	120965521	AAAAT>A	D	5	C5
chr9	41260848	C>CA	I	5	
chr9	133373909	C>CAA	I	5	SURF4
chr9	109452569	C>CT	I	5	PTPN3
chr9	107899568	C>CTG	I	5	
chr9	91053521	CA>C	D	4	LINC02937
chr9	78176680	CT>C	D	5	
chr9	83712444	CT>C	D	5	UBQLN1AS1
chr9	94291047	CTT>C	D	5	ZNF169
chr9	62039845	G>GT	I	5	LOC107987008
chr9	65385550	GCC>G	D	4	
chr9	5930369	TTA>T	D	5	KIAA2026
chrX	103822996	A>AT	I	5	RAB9B
chrX	33918250	C>CT	I	5	LOC105373153
chrX	92033372	C>CT	I	5	PCDH11X
chrX	73031470	C>CTG	I	4	
chrX	100651788	CAA>CA	D	5	SRPX2
chrX	124551528	CCA>C	D	5	TENM1
chrX	100577387	CT>C	D	5	
chrX	137503215	G>GA	I	4	
chrX	150201899	TA>T	D	5	
chrX	137114849	TAC>T	D	5	

Chr. = Chromosome. Mut. = mutation: I = insertion, D = deletion. Change = sequence change in K562-BA sgRNA2 (c-ABL sgRNA1) relative to K562.
